# The Predictive Value of Genetic Analyses in the Diagnosis of Tetrahydrobiopterin (BH4)-Responsiveness in Chinese Phenylalanine Hydroxylase Deficiency Patients

**DOI:** 10.1038/s41598-017-06462-y

**Published:** 2017-07-28

**Authors:** Tianwen Zhu, Jun Ye, Lianshu Han, Wenjuan Qiu, Huiwen Zhang, Lili Liang, Xuefan Gu

**Affiliations:** 1 Department of Endocrinology and Genetic Metabolism, Xin-Hua Hospital, Shanghai Institute of Pediatric Research Affiliated to Shanghai Jiao Tong University School of Medicine, Shanghai, China; 2 Department of Neonatal Medicine, Xin-Hua Hospital Affiliated to Shanghai Jiao Tong University School of Medicine, Shanghai, China

## Abstract

Molecular characterization of PAH deficiency has been proven essential in establishing treatment options. We examine the diagnostic accuracy of two genetic assays to predict BH4 responsiveness: to determine whether the AV sum test or mutation-status assessment test can obviate the need for BH4 loading in Chinese patients. The overall predicted response in 346 patients was 31.65% by the AV sum test and 25.43% by the other assay; both percentages were lower than 51.06% derived from loading results in 94 patients. Responders were compound heterozygotes with definite BH4 responsive mutations, while non-responders had null/null ones; some consistently with specific mutations and genotypes. The sensitivity and specificity of the assays were 81.1% and 92.5% for the AV sum, and 82.9%, 97.3% for the other. An AV sum cutoff >2 has a positive predictive value (PPV) of 90.9%, while the presence of at least one BH4 responsive mutation has a PPV of 97.1%. The two approaches showed good concordance. Our data confirmed that the mutation-status assessment has a higher diagnostic accuracy in predicting response for Chinese patients than the AV sum test. BH4-responsiveness may be predicted or excluded from patients’ molecular characteristics to some extent, thus some patients may avoid the initial loading.

## Introduction

Phenylalanine hydroxylase (PAH; NP_000268, EC 1.14.16.1) deficiency (MIM# 261600) is an autosomal recessive disorder that results from intolerance to the dietary intake of phenylalanine (Phe)^1^. The mainstay of treatment is a Phe-restricted diet^2^, but its life-long application with stable levels between 120 and 360 umol/L^3, 4^ is a challenge that results in high rates of non-compliance and breakdown, which are known to increase with age^5^. Studies^6, 7^ have been conducted to examine the relationship between cerebral complications of the disease and the increase or the fluctuations in Phe concentrations, and it is demonstrated that liberalization of Phe control at any age, especially during the first 10 to 12 years of life^8, 9^, has led to deficits of cognitive and executive functions^10, 11^, impairment of working memory^12^ and other psychological and neurologic abnormalities^13^.

In 2007, the United States Food and Drug Administration (FDA) approved sapropterin dihydrochloride (Kuvan®, formerly known as Phenoptin), a synthetic form of tetrahydrobiopterin (BH4), the first oral pharmacotherapy for PAH deficiency. Two randomized controlled trials^14, 15^ showed that in some patients the drug significantly lowered blood Phe concentration with no serious adverse side-effects and a long term retrospective study^16^ indicated that sapropterin improved their Phe tolerance. Additional research^17, 18^ revealed that sapropterin therapy could increase the stability of blood Phe levels. This group of patients was then classified as ‘tetrahydrobiopterin (BH4)-responsive hyperphenylalaninemia (HPA)/PKU^19^, a new subtype of PAH deficiency.

Therefore, it is of crucial importance to detect this subtype of patients correctly and to determine the individual sensitivity to BH4; a 30% reduction or more in blood Phe concentration 24 hours after administration of sapropterin is the most commonly used biochemical definition of a positive response^20^. Since the corresponding work of the test is onerous, finding a rational testing modality is imperative for further application. As *PAH* gene (NG_008690.1) mutations have been characterized, the association of these mutations with disease phenotypes has been uncovered. It was acknowledged that although different biochemical phenotypes were observed in identical genotypes, their BH4 responsiveness was always the same except in a very small percentage of patients^21–23^, and BH4-responsiveness was a common trait among subjects with the milder forms of PAH deficiency^22, 24, 25^. This observation significantly increased research activities on the feasibility of using genetic analysis to assess the response^26–30^ and a consensus was reached that such research might help to guide clinical decisions and promote personalized medicine in managing PAH deficiency for specific populations.

The prevalence of PAH deficiency in Chinese population is about 1 in 11,572 live births^31^. Previously, our team carried out genetic analyses for patients from diverse regions of China^32, 33^, investigated the mutational spectrum, and established the genotype-phenotype correlations. These studies comprise the initial steps in the development of an optimal molecular diagnostic algorithm. In the current study, we used two assays to predict the prevalence of BH4 response in a cohort of 346 Chinese PAH deficient patients. The assigned values (AV) sum approach^34^ is a genotype severity tool in which the predicted phenotype for a patient was expressed numerically as the sum of two mutations’ AVs. The results for the predicted phenotypes for all our patients were derived from Guldberg *et al*.^34^ (see the Appendix) and our previous paper^32^. The other approach is based on the speculation that the presence of some specific mutations on at least one *PAH* gene copy, which is repeatedly found to be associated with BH4-responsiveness, would be sufficient for positive BH4 response. The performances of both tests in predicting the BH4 response were respectively validated by the clinical BH4 loading results in 94 of them. The accuracy of both analyses in differentiating between BH4 responsive and non-BH4 responsive patients was assessed; whether the AV sum test or mutation-status assessment test can obviate the need for BH4 loading was discerned.

## Results

The 346 PAH deficient patients (Table S1) were classified into three phenotypic categories, 172 (172/346, 49.71%) as classic PKU (cPKU), 156 (156/346, 45.09%) as mild PKU (mPKU), and 18 (18/346, 5.20%) as mild hyperphenylalaninemia (MHP), indicating half of our population as carrying the milder form. All patients displayed 186 different genotypes based on 109 mutations, 53 of which were classified as null^32, 33^. Among the 346 individuals, 35 (35/346, 10.12%) were homozygous [23 for p.Arg243Gln (c.728G > A), 5 for p.Ex6-96A > G (c.611A > G), 3 for p.Arg413Pro (c.1238G > C), 1 for p.Tyr166* (c.498C > A), p.Arg241Cys (c.721C > T), p.Gly257Val (c.770G > T) and p.Leu385Pro (c.1154T > C), respectively], and the remaining patients were compound heterozygotes (311/346, 89.88%). Homozygosity was found in 9.12% of our population and 8 mutations accounted for over two-thirds of the 692 alleles, with p.Arg243Gln (c.728G > A) at a 24.06% frequency. Thus, the investigation of common mutations or allelic combinations in accordance to BH4 responsiveness was of great interest to Chinese patients in general. Based on the AV sum information^32, 34^, 88 out of the 278 individuals (refer to Method: Determining the PAH Genotype Severity Using the AV Sum and Classification of BH4 Response) have an AV sum >2 (88/278, 31.65%), thus being potentially responsive. While for the assessment of the mutation BH4-responsiveness-status, 88 out of the 346 patients (88/346, 25.43%) were potentially responsive.

### BH4 Loading Test

The outcome of the BH4 loading test in 94 patients was summarized in Table 1. Among these patients, 48 (48/94, 51.06%) were shown to be BH4-responders: 5 (5/48, 10.42%) were cPKU, 37 (37/48, 77.08%) were mPKU, and 6 (6/48, 12.50%) were MHP. The non-responders were 46 (46/94, 48.94%): 29 were (29/46, 63.04%) cPKU, 17 (17/46, 39.96%) were mPKU, and none (0/46, 0%) was MHP. It was found that 9 (9/46, 19.57%) of the non-responders had a Phe reduction rate less than 20% at 8 hours after the loading and between 20% and 30% at 24 hours. Fiege B *et al*.^35^ defined this situation as “slow responsiveness”. Interestingly, in our study, these late responsive patients have cPKU (6) and mPKU (3).

**Table 1:** BH4 loading test in 94 Chinese PAH Deficient patients.

Patient ID	Pre-Phe^a^	Phenotype^b^	Protein variation	AV sum^c^	Group^d^	Phe reduction 24 h (%)	BH4 R status^e^	under BH4, Phe became ≤360 umol/L^f^
M914	249	MHP	p.[Arg241Cys]; [Gly247Arg]	?	II	65.63%	R+	yes
M1080	660	mPKU	p.[IVS4-1G > A]; [Arg241Cys]	5	II	87.14%	R+	yes
M1085	469.8	mPKU	p.[Arg241Cys]; [Val388Met]	6	II	85.16%	R+	yes
M1127	258	MHP	p.[Arg241Cys]; [Arg241Cys]	8	II	87.33%	R+	yes
M1229	607.2	mPKU	p.[Arg241Cys]; [IVS7+2 T > A]	5	II	77.30%	R+	yes
M1294	627.6	mPKU	p.[Arg241Cys]; [IVS7+1 G > A]	5	II	92.22%	R+	yes
M1300	600.6	mPKU	p.[Ex6-96 A > G]; [Arg241Cys]	5	II	84.58%	R+	yes
M1314	588	mPKU	p.[Ex6-96 A > G]; [Arg241Cys]	5	II	65.18%	R+	yes
M1343	562.2	mPKU	p.[Arg241Cys]; [; [Leu255Ser]	5	II	83.85%	R+	yes
M1362	451.8	mPKU	p.[Phe161Ser]; [Arg241Cys]	6	II	85.13%	R+	yes
M1374	660	mPKU	p.[Arg241Cys]; [Arg400Lys]	?	II	73.41%	R+	yes
M1451	489	mPKU	p.[Arg111*]; [Arg241Cys]	5	II	87.23%	R+	yes
M1501	1320	cPKU	p.[Arg243Gln]; [IVS12+4 A > G]	2	I	60.24%	R+	yes
M1604	959.4	mPKU	p.[Arg241Cys]; [Arg243Gln]	5	II	58.48%	R+	yes
M1675	510	mPKU	p.[Arg111*]; [Arg241Cys]	5	II	69.52%	R+	yes
M1686	420	mPKU	p.[Arg241Cys]; [Arg243Gln]	5	II	92.04%	R+	yes
M1709	384	mPKU	p.[Ex6-96 A > G]; [Gln419Arg]	5	III	88.70%	R+	yes
M1731	408	mPKU	p.[Ser70del]; [Arg241Cys]	5	II	60.09%	R+	Yes
M1772	228	MHP	p.[Arg155His]; [Val399Val]	5	III	79.15%	R+	yes
M1837	924	mPKU	p.[Ex6-96 A > G]; [Arg241Cys]	5	II	68.90%	R+	yes
M1853	822	mPKU	p.[Ser70del]; [Arg158Gln]	2	I	72.51%	R+	yes
M1950	342	MHP	p.[Ex6-96 A > G]; [Arg241Cys]	5	II	75.20%	R+	yes
M810	849	mPKU	p.[Arg241Cys]; [Arg413Pro]	5	II	82.91%	R+	yes
M835	833.4	mPKU	p.[Arg241Cys]; [IVS7+2 T > A]	5	II	54.26%	R+	yes
M840	780	mPKU	p.[Ser70del]; [Val388Met]	3	II	46.79%	R+	yes
M855	780	mPKU	p.[Arg243Gln]; [Ile324Asn]	?	III	47.62%	R+	yes
M862	360	MHP	p.[Ex6-96 A > G]; [Arg241Cys]	5	II	79.67%	R+	yes
M865	900	mPKU	p.[IVS4-1G > A]; [721 C > T]	5	II	99.75%	R+	yes
M870	330	MHP	p.[Ser70del]; [Arg261Gln]	3	II	72.57%	R+	yes
M878	960	mPKU	p.[Ser70del]; [Arg243Gln]	2	I	52.21%	R+	yes
M882	804	mPKU	p.[Glu183Gly]; [Arg241Cys]	?	II	53.33%	R+	yes
M896	660	cPKU	p.[Arg241Cys]; [Glu286Lys]	?	II	80.34%	R+	yes
M904	534	mPKU	p.[Phe121Leu]; [Arg241Cys]	?	II	89.62%	R+	yes
M915	1680	cPKU	p.[IVS4-1G > A]; [Tyr356*]	2	I	56.96%	R+	yes
M922	627	mPKU	p.[Arg243Gln]; [Met276Lys]	?	III	64.73%	R+	yes
M952	576	mPKU	p.[Arg241Cys]; [Glu286Lys]	?	II	85.74%	R+	yes
M987	552	mPKU	p.[Arg241Cys]; [Gly247Arg]	?	II	53.67%	R+	yes
M1105	616.8	mPKU	p.[Arg241Cys]; [Arg243Gln]	5	II	32.25%	R+	no
M1437	1020	mPKU	p.[Pro275Ala]; [Tyr356*]	?	III	36.72%	R+	no
M1480	720	mPKU	p.[Arg243Gln]; [Arg261Gln]	3	II	42.26%	R+	no
M1483	1260	cPKU	p.[Arg243Gln]; [Val399Val]	2	I	30.12%	R+	no
M1785	1980	cKU	p.[Ex6-96 A > G]; [Val388Met]	3	II	34.24%	R+	no
M1829	880.8	mPKU	p.[Phe161Ser]; [Val399Val]	3	III	31.03%	R+	no
M793	1740	cPKU	p.[Ex6-96 A > G]; [Arg243Gln]	2	I	39.22%	R+	no
M857	938.4	mPKU	p.[Arg243Gln]; [Val388Met]	3	II	39.55%	R+	no
M1334	1080	mPKU	p.[Arg243Gln]; [Gly247Val]	2	I	34.17%	R+	no
M1340	1080	mPKU	p.[Arg243Gln]; [Ala434Asp]	3	III	37.42%	R+	no
M996	894	mPKU	p.[Ala156Pro]; [Arg408Gln]	?	II	58.49%	R+	no
M1268	1830	cPKU	p.[Arg243Gln]; [Cys357*]	2	I	12.79%	R−	no
M1269	2057.4	cPKU	p.[Arg111*]; [Arg413Pro]	2	I	9.24%	R−	no
M1304	1500	cPKU	p.[Tyr166*]; [Ex6-96 A > G]	2	I	25.01%	R−	no
M1438	1320	cPKU	p.[IVS4-1G > A]; [Arg413Pro]	2	I	0.42%	R−	no
M1463	510	mPKU	p.[Arg111*]; [Val399Val]	2	I	17.00%	R−	no
M1516	1020	mPKU	p.[Phe161Ser]; [Arg243Gln]	3	I	21.82%	R−	no
M1518	1296	cPKU	p.[Arg111*]; [Met276Arg]	?	III	0.00%	R−	no
M1565	1680	cPKU	p.[Arg243Gln]; [Arg243Gln]	2	I	15.83%	R−	no
M1575	2400	cPKU	p.[Arg111*]; [Arg243Gln]	2	I	26.46%	R−	no
M1590	1373.4	cPKU	p.[Ex6-96 A > G]; [Arg243Gln]	2	I	5.93%	R−	no
M1623	1108.2	mPKU	p.[Arg243Gln]; [Arg243Gln]	2	I	11.19%	R−	no
M1630	1920	cPKU	p.[Arg243Gln]; [Tyr356*]	2	I	−0.48%	R−	no
M1649	900	mPKU	p.[Arg261*]; [Tyr356*]	2	I	−20.00%	R−	no
M1651	1500	cPKU	p.[Arg243Gln]; [Ala434Asp]	3	III	12.74%	R−	no
M1660	816	mPKU	p.[Arg243Gln]; [Arg243Gln]	2	I	8.77%	R−	no
M1674	500.4	mPKU	p.[Tyr356*]; [Ala434Asp]	3	III	8.86%	R−	no
M1710	931.2	mPKU	p.[Ex6-96 A > G]; [Arg413Pro]	2	I	3.71%	R−	no
M1714	1506	cPKU	p.[Arg111*]; [Tyr356*]	2	I	1.73%	R−	no
M1733	1071	mPKU	p.[Arg243Gln]; [Val399Val]	2	I	8.59%	R−	no
M1744	1440	cPKU	p.[Ex6-96 A > G]; [Arg243Gln]	2	I	16.52%	R−	no
M1747	1504.8	cPKU	p.[Gln232*]; [Arg243Gln]	2	I	10.29%	R−	no
M1753	694.2	mPKU	p.[Ex6-96 A > G]; [Tyr356*]	2	I	11.67%	R−	no
M1755	1115.4	mPKU	p.[Ex6-96 A > G]; [Arg243Gln]	2	I	9.59%	R−	no
M1770	1651.8	cPKU	p.[IVS4-1G > A]; [Ile406Thr]	?	III	5.81%	R−	no
M1775	1335	cPKU	p.[Arg243Gln]; [Arg243Gln]	2	I	29.54%	R−	no
M1782	1218	cPKU	p.[IVS4-1G > A]; [Arg243Gln]	2	I	20.44%	R−	no
M1799	1141.2	mPKU	p.[Arg111*]; [Tyr356*]	2	I	0.77%	R−	no
M1820	666	mPKU	p.[Arg243Gln]; [Arg252Gln]	2	III	13.24%	R−	no
M1846	1800	cPKU	p.[Ex6-96 A > G]; [Arg243Gln]	2	I	13.54%	R−	no
M1891	1260	cPKU	p.[Ex6-96 A > G]; [Arg243Gln]	2	I	0.00%	R−	no
M1916	1320	cPKU	p.[His64*]; [Ala156Pro]	?	III	12.07%	R−	no
M1952	624	mPKU	p.[Arg111*]; [Arg243Gln]	2	I	0.00%	R−	no
M744	2100	cPKU	p.[Arg243Gln]; [Arg243Gln]	2	I	2.00%	R−	no
M899	2040	cPKU	p.[Ex6-96 A > G]; [IVS6-1G > A]	2	I	22.58%	R−	no
M908	660	mPKU	p.[Glu280Lys]; [Ala434Asp]	3	III	27.46%	R−	no
M911	1734	cPKU	p.[Arg158Trp]; [Val399Val]	?	III	10.71%	R−	no
M920	1006.2	mPKU	p.[Gly257Val]; [Arg408Gln]	?	II	15.86%	R−	no
M936	1920	cPKU	p.[IVS4-1G > A]; [Arg413Pro]	2	I	24.32%	R−	no
M960	1260	cPKU	p.[Ex6-96 A > G]; [Arg243Gln]	2	I	0.00%	R−	no
M1316	876	mPKU	p.[Ex6-96 A > G]; [Arg243Gln]	2	I	29.40%	R−	no
M1339	1920	cPKU	p.[Arg413Pro]; [Arg413Pro]	2	I	11.89%	R−	no
M1342	726.6	mPKU	p.[Gly247Val]; [Cys357Tyr]	?	III	2.22%	R−	no
M1349	1218	cPKU	p.[Arg111*]; [Val399Val]	2	I	19.51%	R−	no
M1401	564	mPKU	p.[Ex6-96 A > G]; [Ex6-96 A > G]	2	I	−44.77%	R−	no
M1412	1260	cPKU	p.[Ex6-96 A > G]; [Arg261*]	2	I	9.34%	R−	no
M1420	1260	cPKU	p.[Arg176*]; [IVS6-1G > A]	2	I	15.33%	R−	no

^a^The highest plasma Phe concentration (umol/L)before treatment, and all of which applied in this study were the maximum pretreatment values.

^b^Phenotypes were stratified as mild hyperphenylalaninemia (MHP), mild phenylketonuria (mPKU), and classical PKU (cPKU) based on the pretreatment plasma Phe values.

^c^An arbitrary value (AV) if available was assigned to each mutation: AV = 1 for classical PKU mutation; AV = 2 for moderate PKU mutation; AV = 4 for mild PKU mutation, and AV = 8 for MHP mutation. Phenotypes resulting from a combination of the two mutant alleles were expressed as the sum of the two mutations’ AVs.

^d^The groups for the patients based on the assessment of the BH4-responsiveness-status of the detected mutations. I for “Non BH4 responsive”; II for “BH4 responsive”; III for “Undefined BH4 responsive”.

^e^Based on BH4 loading test; R: response; R+: responder; R−: non-responder.

**Figure 1:**
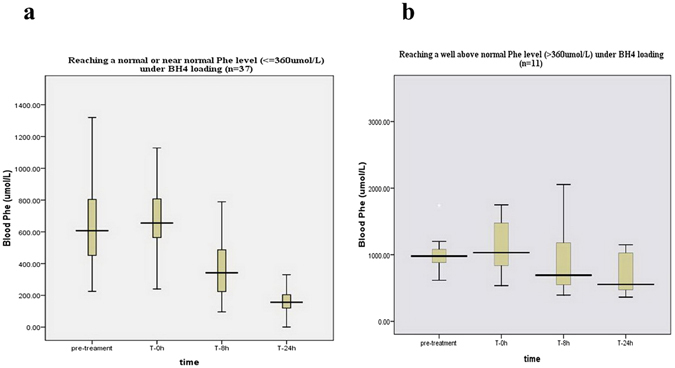
The length of the boxes indicates the interquartile space (P25–P75); the horizontal line into the box represents the median (P50), and the error bars indicate the adjacent values, that is, the maximum and minimum values of the distribution, which may not be considered abnormal.

According to the phenotypic scheme, response to BH4 was detected in 15.15% (5/33) of cPKU, in 67.27% (37/55) of mPKU and in all the 6 MHP patients. The differences in the percentages of responders among the three phenotypic groups were statistically significant (χ2 = 28.56, df = 2, p < 0.001). As shown in Table 1, the reduction of Phe from the baseline among BH4 responders ranged between 30.12% and 99.75%, revealing a considerable degree of inter-subject variability. Most patients (37/48, 77.08%) had normal or nearly normal (≤360 umol/L) Phe levels 24 hours following the BH4 challenge whereas 11 responders had not. The Phe for 3 out of 5 cPKU responders and 28 out of 37 mPKU responders reached the normal range while all 6 MHP patients showed pronounced Phe reductions, indicating that the rate of Phe reduction was largely dependent on the phenotypic class of the patients. For the group of 37 responders (Fig. 1a), blood Phe level was 642.32 ± 294.23 umol/L during pre-treatment and 688.65 ± 216.23 umol/L at time 0 hour of BH4 loading. The latter number was reduced to 170.62 ± 83.80 umol/L at 24 hours after loading. Data analysis of this group suggested a significant correlation between pre-treatment Phe and the percentage of Phe reduction after challenge (r = 0.378, P < 0.05) as opposed to before-BH4 (time at 0 hour) Phe and the extent of response which did not seem to have a statistically significant correlation (r = 0.08, P > 0.05) (The statistical correlation between pre-treatment Phe and before-BH4 (time at 0 hour) was excluded (r = −0.209, P > 0.05)). The group of the other 11 responders (Fig. 1b) had a pre-treatment Phe concentration of 1110.00 ± 413.54 umol/L and 1238.82 ± 710.33 umol/L at 0 hour of BH4 loading. The latter number was reduced to 733.07 ± 454.64 umol/L at the end of the test. Significant correlations existed between pre-treatment Phe and post-loading Phe level (time at 24 hour) (r = 0.777, P < 0.01), and between pre-BH4 Phe and Phe level at 24 hours (r = 0.981, P < 0.001), which was inconsistent with the view derived from the study of Leuzzi, V. *et al*.^36^.

### Classification of Mutations and Genotypes for BH4 Response

Genetic analysis revealed 45 mutations and identified 66 genotypes in these 94 patients (Table 2). Among 43 patients with 2 pre-defined null mutations, 7 patients were identified as responders: p.[Arg243Gln]; [Gly247Val], p.[Arg243Gln]; [IVS12 + 4A > G], p.[Arg243Gln]; [Val399Val], p.[Ser70del]; [Arg158Gln], p.[Ex6-96A > G]; [Arg243Gln], p.[Ser70del]; [Arg243Gln], p.[IVS4-1G > A]; [Tyr356*]. For 9 mutations involved, the intrinsic BH4 responsiveness might be ambiguous due to category overlap. Among the 35 patients presenting at least 1 mutation that had been reported to associate with BH4 responsiveness, only 1 patient, i.e. p.[Gly257Val]; [Arg408Gln], did not respond to the challenge. Among 16 patients with the presence of mutations without definite BH4 responsiveness, all of them were functionally hemizygous which could help us to assess the intrinsic BH4 responsiveness of the missense mutation present on the other chromosome. From the derivation of our data, we classified p.Arg241Cys (c.721C > T), p.Val388Met (c.1162G > A), and p.Arg261Gln (c.782G > A) as BH4 responsive mutations, p.Arg111* (c.331C > T), p.[IVS6-1G > A] (c.707-1G > A) and p.Arg413Pro (c.1238G > C) as constantly non-BH4 responsive variants. Both classifications were unambiguous because the response was consistent in two or more functionally hemizygous patients. Since p.Arg408Gln (c. 1223G > A) was found in two compound-heterozygous patients, and the following mutations including p.Arg155His (c.464G > A), p.Arg158Gln (c.473G > A), p.Pro275Ala (c.823C > G), p.Met276Lys (c.827T > A), p.Ile324Asn (c.971T > A) and p.Gln419Arg (c.1256A > G) were found in only one functionally hemizygous patient, the assignment of these variants to any specific BH4 responsive category may therefore be uncertain.

**Table 2:** Genotype versus BH4 response in 94 Chinese PAH Deficient patients.

Genotype^a^	No	responder	non-responder	Inconsistencies (No.)
**null/null**				
p.[Arg243Gln]; [IVS12 + 4 A > G]	1	1cPKU	0	1
p.[Ex6-96 A > G]; [Arg243Gln]	8	1cPKU	5cPKU/2mPKU	1
p.[IVS4-1G > A]; [Tyr356*]	1	1cPKU	0	1
p.[Arg243Gln]; [Gly247Val]	1	1mPKU	0	1
p.[Arg243Gln]; [Val399Val]	2	1mPKU	1mPKU	1
p.[Phe161Ser]; [Arg243Gln]	1	1mPKU	0	1
p.[Ser70del]; [Arg243Gln]	1	1mPKU	0	1
p.[Arg243Gln]; [Cys357*]	1	0	1cPKU	0
p.[Arg111*]; [Arg413Pro]	1	0	1cPKU	0
p.[Tyr166*]; [Ex6-96 A > G]	1	0	1cPKU	0
p.[Arg413Pro]; [Arg413Pro]	1	0	1cPKU	0
p.[Ex6-96 A > G]; [Arg261*]	1	0	1cPKU	0
p.[Arg176*]; [IVS6-1G > A]	1	0	1cPKU	0
p.[IVS4-1G > A]; [Arg413Pro]	2	0	2cPKU	0
p.[Arg243Gln]; [Arg243Gln]	5	0	3cPKU/2mPKU	0
p.[Arg111*]; [Arg243Gln]	2	0	1cPKU/1mPKU	0
p.[Arg243Gln]; [Tyr356*]	1	0	1cPKU	0
p.[Arg111*]; [Tyr356*]	2	0	1cPKU/1mPKU	0
p.[Gln232*]; [Arg243Gln]	1	0	1cPKU	0
p.[IVS4-1G > A]; [Arg243Gln]	1	0	1cPKU	0
p.[Ex6-96 A > G]; [IVS6-1G > A]	1	0	1cPKU	0
p.[Arg158Trp]; [Val399Val]	1	0	1cPKU	0
p.[Arg111*]; [Val399Val]	2	0	1cPKU/1mPKU	0
p.[Arg261*]; [Tyr356*]	1	0	1mPKU	0
p.[Ex6-96 A > G]; [Arg413Pro]	1	0	1mPKU	0
p.[Ex6-96 A > G]; [Tyr356*]	1	0	1mPKU	0
p.[Ex6-96 A > G]; [Ex6-96 A > G]	1	0	1mPKU	0
**Tota**l	43	7	36	7
**at least one mutation with definite BH4 responsiveness**				
p.[Ex6-96 A > G]; [Val388Met]	1	1cPKU	0	0
p.[IVS4-1G > A]; [Arg241Cys]	2	2mPKU	0	0
p.[Arg241Cys]; [Arg243Gln]	3	3mPKU	0	0
p.[Arg241Cys]; [IVS7 + 2 T > A]	2	2mPKU	0	0
p.[Arg241Cys]; [IVS7 + 1 G > A]	1	1mPKU	0	0
p.[Ex6-96 A > G]; [Arg241Cys]	5	3mPKU/2MHP	0	0
p.[Arg241Cys]; [Arg400Lys]	1	1mPKU	0	0
p.[Arg243Gln]; [Arg261Gln]	1	1mPKU	0	0
p.[Ser70del]; [Val388Met]	1	1mPKU	0	0
p.[Arg243Gln]; [Val388Met]	1	1mPKU	0	0
p.[Glu183Gly]; [Arg241Cys]	1	1mPKU	0	0
p.[Arg241Cys]; [Glu286Lys]	2	2mPKU	0	0
p.[Ala156Pro]; [Arg408Gln]	1	1mPKU	0	0
p.[Arg241Cys]; [Val388Met]	1	1mPKU	0	0
p.[Arg241Cys]; [Arg241Cys]	1	1MHP	0	0
p.[Arg241Cys]; [Leu255Ser]	1	1mPKU	0	0
p.[Phe161Ser]; [Arg241Cys]	1	1mPKU	0	0
p.[Arg111*]; [Arg241Cys]	2	2mPKU	0	0
p.[Ser70del]; [Arg241Cys]	1	1mPKU	0	0
p.[Arg241Cys]; [Arg413Pro]	1	1mPKU	0	0
p.[Ser70del]; [Arg261Gln]	1	1MHP	0	0
p.[Phe121Leu]; [Arg241Cys]	1	1mPKU	0	0
p.[Arg241Cys]; [Gly247Arg]	2	1mPKU/1MHP	0	0
p.[Gly257Val]; [Arg408Gln]	1	0	1mPKU	1
**Total**	35	34	1	1
**mutations without definite BH4 responsiveness**				
p.[Arg243Gln]; [Ala434Asp]	2	1mPKU	1cPKU	/
p.[Ser70del]; [Arg158Gln]	1	1mPKU	0	/
p.[Ex6-96 A > G]; [Gln419Arg]	1	1mPKU	0	/
p.[Arg155His]; [Val399Val]	1	1MHP	0	/
p.[IVS4-1G > A]; [Ile406Thr]	1	0	1cPKU	/
p.[His64*]; [Ala156Pro]	1	0	1cPKU	/
p.[Gly247Val]; [Cys357Tyr]	1	0	1mPKU	/
p.[Phe161Ser]; [Arg243Gln]	1	0	1mPKU	/
p.[Arg111*]; [Met276Arg]	1	0	1mPKU	/
p.[Arg243Gln]; [Arg252Gln]	1	0	1mPKU	/
p.[Glu280Lys]; [Ala434Asp]	1	0	1mPKU	/
p.[Tyr356*]; [Ala434Asp]	1	0	1mPKU	/
p.[Pro275Ala]; [Tyr356*]	1	1mPKU	0	/
p.[Arg243Gln]; [Ile324Asn]	1	1mPKU	0	/
p.[Arg243Gln]; [Met276Lys]	1	1mPKU	0	/
**Total**	16	7	9	

^a^The genotypes are described at protein level.

Table 2 presents the 66 different genotypes identified in these patients: 34 different genotypes belonging to the responder group, 29 belonging to the non-responder group, and 3 belonging to both groups. Genotypes occurring in two or more patients showed consistency in BH4 loading results: p.[IVS4-1G > A]; [Arg241Cys], p.[Arg241Cys]; [Arg243Gln], p.[Arg241Cys]; [IVS7 + T > A], p.[Ex6-96A > G]; [Arg241Cys], p.[Arg241Cys]; [Glu286Lys], p.[Arg111*]; [Arg241Cys] and p.[Arg241Cys]; [Gly247Arg] in responders; p.[Arg243Gln]; [Arg243Gln], p.[IVS4-1G > A]; [Arg413Pro], p.[Arg111*]; [Arg243Gln], p.[Arg111*]; [Tyr356*] and p.[Arg111*]; [Val399Val] in non-responders; p. [Ex6-96A > G]; [Arg243Gln], p.[Arg243Gln]; [Ala434Asp] and p.[Arg243Gln]; [Val399Val] belonged to both groups.

### The Diagnostic Performance of PAH Genotype AV Sum to Predict BH4 Response

The *PAH* genotypes, AV sums, and clinical BH4 responsive status of 77 patients (refer to Method: Determining the PAH Genotype Severity Using the AV Sum and Classification of BH4 Response) were shown in Table 1. The majority of responders (81.08%, 30/37) had an AV sum >2, indicating that at least one mutation is moderate or mild in severity. The remaining seven responders carried two severe mutations (AV = 1) and were given an AV sum of 2. BH4 responders represented a specific heterogeneous group, with the AV sum ranging from 2 to 8, 8 being the highest AV sum present in a homozygote for p.Arg241Cys. In contrast, the AV sum for the majority of non-responders (92.5%, 37/40) was 2, indicating a severe *PAH* genotype. The other three non-responders who carried a moderate mutation in combination with a severe one had an AV sum of 3.

**Figure 2:**
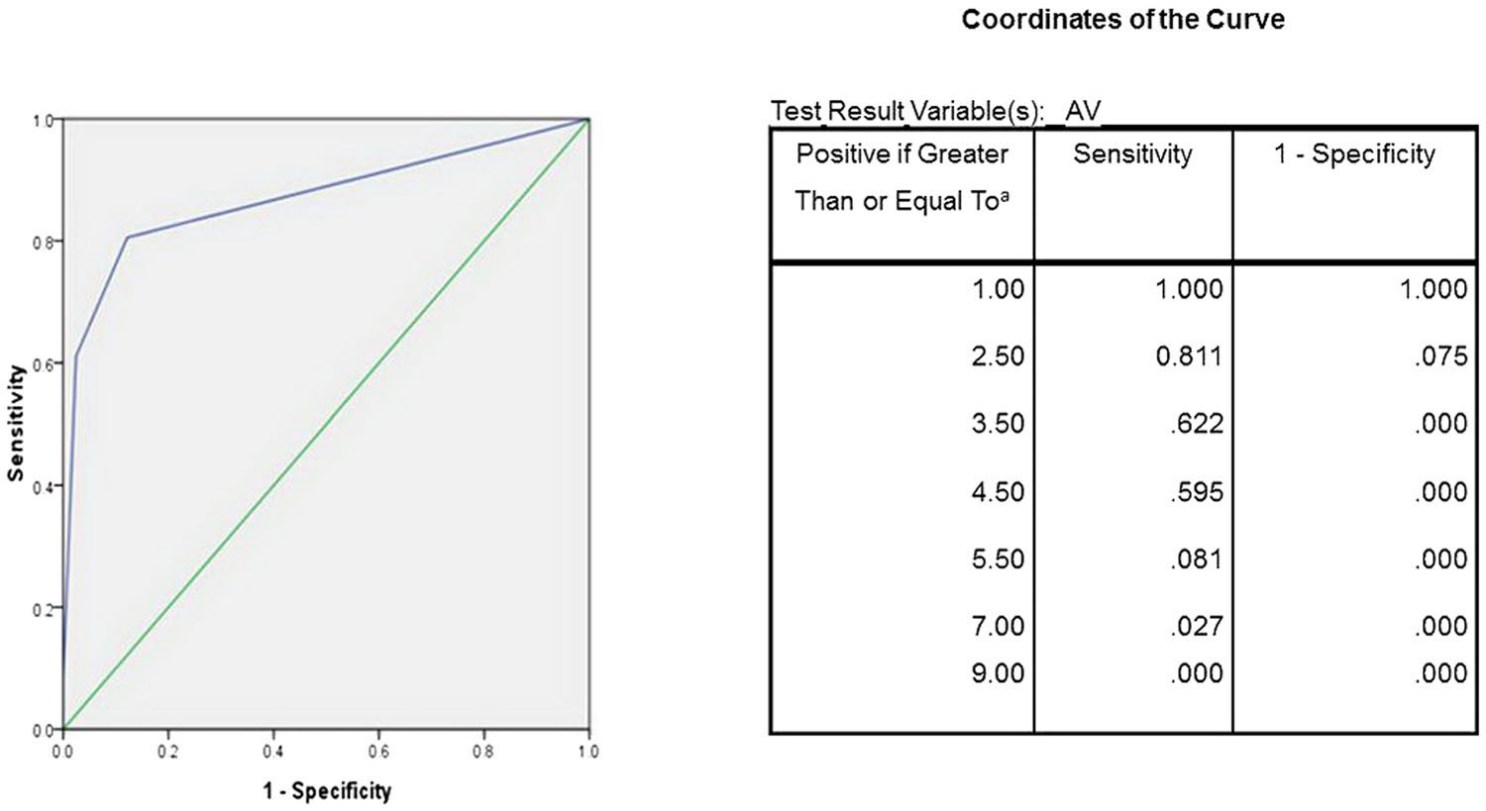
The test result variable(s): AV has at least one tie between the positive actual state group and the negative actual state group. *The smallest cutoff value is the minimum observed test value minus 1, and the largest cutoff value is the maximum observed test value plus 1. All the other cutoff values are the averages of two consecutive ordered observed test values.

Then, we analyzed ROC curve (Fig. 2) to verify the efficiency of this approach and to determine the cutoff value of the AV sum that differentiated PAH deficient patients with and without BH4 response. The AUC value was 0.891 (95% confidence interval (95% CI) = 0.811–0.972)). The optimal cutoff level of the AV sum, determined by the curve to calculate sensitivity and specificity, was set at 2.5. Since AV sums ranged from 2 to 8, with a whole number indicating the patient phenotype, we used AV sum >2 as the cutoff. The sensitivity and specificity we found at this level was 81.1% and 92.5%, which likely resulted in a positive predictive value (PPV) as opposed to a negative predictive value (NPV) of 90.9% and 84.1% (Table 3).

**Table 3:** Diagnostic performance of two predicted tests, estimated using the BH4 loading as the gold standard.

	Sensitivity (%)	Specificity (%)	PPV (%)	NPV (%)	Accuracy (%)
AV sum (cut-off: >2)	81.1%	92.5%	90.9%	84.1%	87.01%
BH4-responsiveness-status of the mutations	82.9%	97.3%	97.1%	83.7%	89.74%

PPV: positive predictive value; NPV: negative predictive value; OP: odd product; A: true positive (TP); B: false negative (FN); C: false positive (FP); D: true negative (TN).

Sensitivity = True positive rate (TPR) = A/(A + B) = TP/(TP + FN).

Specificity = True negative rate (TNR) = D/(C + D) = TN/(FP + TN).

PPV = precision = A/(A + C) = TP/(TP + FP).

NPV = TN/(TN + FN).

Accuracy = (A + D)/(A + B + C + D).

### The Diagnostic Performance of Assessment of the Mutation BH4-Responsiveness-Status to Predict BH4 Response

Based on the BH4-responsiveness-status of every mutation, in this cohort of 94 patients, 43 patients carried known null mutations on both alleles, excluding BH4 responsiveness in 45.74% (43/94); 35 patients carried at least one reported mutation with consistent BH4 responsiveness, indicating a high probability of BH4 responsiveness in 37.23% (35/94); and the remaining 16 patients without defined BH4 responsive mutations (Table 2). We note that the observed phenotype of the patient did not always match his predicted phenotype. As shown in Table 2, the observed BH4 response/non-response results matched that of the expected in 70 out of 78 patients (89.74%, 70/78), and the discrepancies were most apparent in patients with expected non-BH4 responsiveness (16.28%, 7/43).

Table 3 shows the diagnostic accuracy of this approach in predicting PAH deficient patients’ clinical BH4 response status. The sensitivity, specificity, PPV and NPV related to the presence of a BH4 responsive variant were 82.9%, 97.3%, 97.1% and 83.7% respectively.

Finally, we used Kappa test to examine the consistency between two approaches in classification of the clinical BH4 response status. Kappa test revealed that the results of the AV sum approach showed good consistency with those of the mutation BH4-responsiveness-status assay (Kappa test, kappa = 0.71, P < 0.001).

## Discussion

Sapropterin has been available as a non-dietary therapy option for patients with BH4-responsive PKU since 2007 in the United States, 2008 in the European Union, and 2010 in Canada. It is prescribed to patients 4 years of age and onwards^4^. The implementation of pharmacological intervention at a younger age has been recently indicated to be feasible and beneficial^37^. It is also noted that sapropterin could be considered as a treatment option in pregnant women with PKU who cannot achieve the recommended ranges of blood Phe level with dietary therapy alone^38^. So a careful selection of patients who are eligible for the therapy using sound procedures (with low false-negatives and false-positives) is imperative. The goal of this study is to investigate the characteristics of BH4-responsiveness in Chinese patients and to determine the predictive value of genetic analysis for BH4-responsiveness in order to identify the appropriate candidates for this medication.

Our BH4 loading results demonstrated that BH4 responsiveness was mostly associated with milder phenotypes (43/48, 89.58%) except 5 (5/48, 10.42%) BH4 responders being cPKU, and the rate of Phe reduction was largely dependent on phenotypic classes with 28 mPKU responders, 6 MHP responders and 3 cPKU responders reaching normal or nearly normal (≤360 umol/L) Phe levels 24 hours post load. These findings supported the statement that mild phenotypes respond better to BH4 challenge^22^. Therefore, we speculated that half our patients (50.29%, 174/346) might be potential candidates for future pharmacologic intervention with concomitant relief or withdrawal of the burdensome diet therapy. In our group, we noted that there were individuals (8/18, 44.44%) who were previously untreated and in whom a positive response to BH4 was identified. The results of our study, coupled with the findings proposed by Moseley KD^39^, Koch R^40^ and Grosse SD^41^, led to the consideration that sapropterin should be an appropriate treatment option for some Chinese patients, and that it might be useful as an adjuvant in maintaining blood Phe levels, thus hindering fluctuations. Inhibiting the fluctuations in blood Phe levels has been recognized as neuro-protective^42^, particularly during infancy, early childhood, and pregnancy. Moreover, sapropterin may allow BH4-responsive patients, who were later diagnosed, to make progress in their development.

Our previous work^32^ has proven that genetic diagnosis was useful for evaluating the biochemical phenotypes. In this study, we estimated the accuracy of two commonly used genetic diagnostic tests for their statistical prediction of BH4 responsiveness. The sensitivity and specificity relative to the BH4 responsiveness status of the detected mutations in predicting clinical response were 82.9% and 97.3%, respectively, a little higher than those of the AV sum tool, 81.1% and 92.5%. Our results showed that the AV sum approach appeared to provide a lesser degree of sensitivity for identifying patients, which was not consistent with the view proposed by Quirk *et al*.^43^, who concluded that the AV sum tool was viable for identifying definitive responders with a predictive sensitivity of 89.5%, as derived from their clinical results of a cohort of 58 patients.

Furthermore, we evaluated the effects of the two tests in 346 Chinese patients. Our data identified 31.65% of our cohort as potentially BH4-responsive using the AV sum test and 25.43% by the other. But the overall frequency of BH4-responsiveness in 94 patients who took the loading test was 51.06%, almost two times higher than the predicted data. The following reasons might contribute to this discrepancy. In our study, only p.Arg241Cys (c.721C > T), p.Val388Met (c.1162G > A) and p.Arg261Gln (c.782G > A) were confirmed to be constantly responsive; p.Arg243Gln (c.728G > A) and p.Arg413Pro (c.1238G > C) which were defined as responsive by Zurfluh *et al*.^22^, were defined as null mutations. Apart from the different criteria for mutation classification due to population-based findings, a unique genotypic feature of high heterogeneity was partly related to it. The mutation spectrum observed in China^33^ showed a characteristic of the combination of a small number of common mutations and a very high number of rare ones. Based on our observation, for the most common 8 mutations, p.Arg241Cys (c.721C > T) was consistently associated with BH4 responsiveness; p.Arg243Gln (c.728G > A) was associated with non-BH4 responsiveness except for 5 patients; p.Ex6-96A > G (c.611A > G), p.[IVS4-1G > A] (c.442-1G > A), and p.Val399Val (c.1197A > T) as splicing mutations, were equally distributed in both categories; while p.Arg413Pro (c.1238G > C), p.Arg111* (c331C > T) and p.[IVS6-1G > A] (c. 707-1G > A) were consistent with their non-BH4 responsiveness in every patient, which might be the reason that the lower “concordance rate” (83.72%) was observed in patients within the expected non-BH4 responsive group. In our study, the patients with the rare mutations were excluded from the AV sum analysis or were assigned to the “Undefined BH4 responsive” group since most rare mutations had not been analyzed *in vitro* and were of unknown BH4 responsiveness.

In terms of optimizing the use and allocation of public health resources, the BH4 response detection procedure based on genotyping is an imperative step for our population since there is an estimated not-too-low potential incidence of BH4-responsiveness in China, yet the drug used for treatment is of high cost with a limited supply. We will present detailed reasons below: first, a genotype-predicted prevalence of BH4-responsiveness is almost two times lower than the result obtained by loading tests, this may lead to lower false positive results, thus eliminating the need for challenging every patient; second, the information of established BH4 responsiveness or non-responsiveness in single allele and mutational combinations derived from our research allows Chinese patients with those genotypes to avoid prior 24 h BH4 loading tests; third, since up to more than 10% of classic patients responded to loading test, 32.73% of mild ones did not respond, and 19.57% of the non-responders were “slow responsive” based on our data, these patients may need a longer period of time for testing to show observable BH4 long-term efficiency; the response in these patients is a difficult target to evaluate properly and the current BH4 loading test should be optimized accordingly to determine the patients who are feasible subjects for complete diet liberalization or who can increase their intake of natural protein with sapropterin as an adjuvant option.

**Figure 3:**
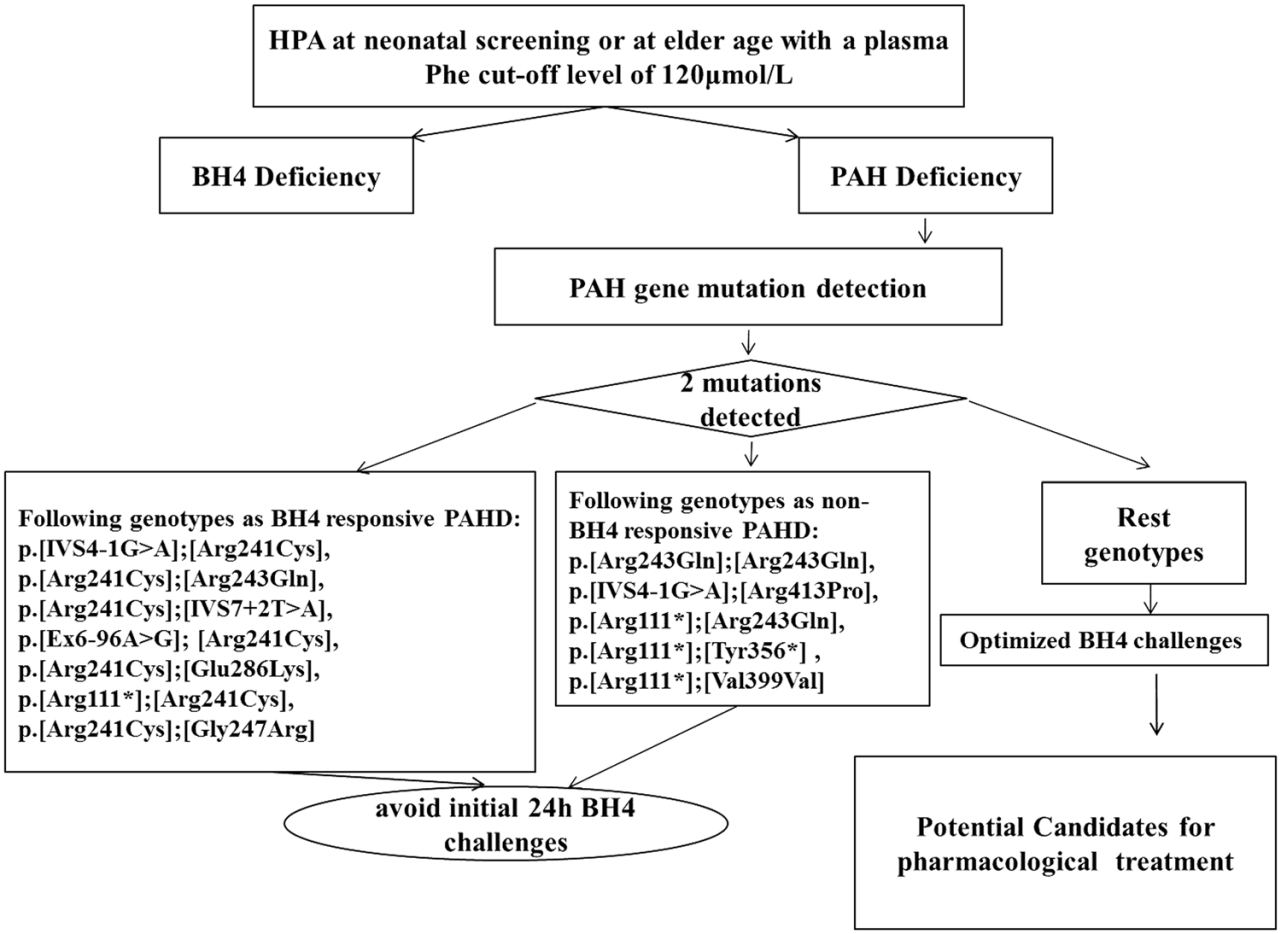
HPA: hyperphenylalaninemia; BH4 deficiency: genetic defects in the path way of BH4 synthesis or recycling leading to secondary PAH deficiency and elevated blood Phe levels; PAH deficiency: the inborn error of Phe metabolism caused by deficient activity of phenylalanine hydroxylate; Initial 24 h BH4 challenges: a BH4-loading test with a dose of 20 mg/kg body weight and duration of 24 h; Optimized BH4 challenges: more personalized testing procedures with different dosage and duration to address individual BH4 responsiveness in PAH deficient patients.

In this way, the inconsistency of our work and the genetic analysis importance in Chinese patients stressed the need for a population-specialized approach to the evaluation of BH4 responsiveness. As a next step, we aimed to verify the clinical relevance of our personalized procedure (Fig. 3) and to analyze the effects of different sapropterin concentrations, dosages, and durations on the outcome of BH4 loading tests in individuals with different genotypes in a prospective study, which might serve as a way to establish individualized treatment for Chinese PAH deficiency patients.

## Methods

### Population Study

Our study included 346 unrelated Chinese patients (171 males and 175 females, with ages ranging from 1 month to 13 years) from the Department of Pediatric Endocrinology and Genetic Metabolism of Xin Hua Hospital affiliated with Shanghai Jiao Tong University School of Medicine between January, 2006 and December, 2012 (Table S1). These patients were found to have two presumably causative mutations in the *PAH* gene. In all patients, PAH deficiency had been detected by either a national screening (56.94%, 197/346) or by the presence of neurological deterioration observed at an older age (43.06%, 149/346) with a plasma Phe cutoff level of 120 μmol/L. Patients with defect in the synthesis and recycling of BH4 were excluded by analysis of urinary pterins and dihydropteridine reductase activity in erythrocytes. Of these patients, 94 whose informed consents were obtained took part in BH4 loading test (40 males and 54 females, ages ranging from 1 month to 5.6 years; 18 patients presented neurological deterioration at an older age while the others were detected with a national screening). Table S2 showed that there were no significant differences between the demographic characteristics of patients who took the BH4 loading tests and those who did not.

The metabolic phenotype for each patient was stratified according to the individual’s plasma Phe concentration before treatment. All of the Phe levels were maximum values (Phe range of 132–2937 umol/L) during pretreatment. Phe was measured using a fluorometric method until 2003 followed by tandem-mass spectrometry. Patients were classified as cPKU (Phe more than 1200 μmol/L), mPKU (Phe, 360 to 1200 μmol/L), and MHP (Phe less than 360 μmol/L) according to the Chinese HPA consensus updated in 2014^3^.

The Ethics Committee of Xinhua Hospital affiliated with Shanghai Jiao Tong University School of Medicine approved this study and informed consents were subsequently obtained from the parents of the patients enrolled. The methods were performed in accordance with the relevant approved guidelines.

### BH4 Loading Test

The BH4 loading tests that were used as the gold standard diagnostic method for BH4 response in this study were performed between 2005 and 2009 following the routine protocol^20^ combined with our local regulation^44^. During the entire testing period, patients had no dietary restrictions. Blood Phe was measured by dried blood spot analysis at times 0-2-4-6-8 and 24 hours after oral administration of 20 mg/kg KUVAN™ (sapropterin dihydrochloride); Biomarin Pharmaceutical Inc. Novato, CA) in 68 patients whose initial plasma Phe results were ≥600 umol/L. For 26 patients whose Phe levels were <600 umol/L, L-Phe (100 mg/kg; Shanghai Pujiang Institute of Applied Biochemistry, China) was given initially, followed by sapropterin (20 mg/kg). Patients’ blood samples were taken at times 0-1-2 and 3 hours after Phe loading and at 0-2-4-6-8 and 24 hours after BH4 loading. Responsiveness to BH4 was calculated as a percentage of blood Phe reduction 24 hours after sapropterin administration. A reduction of at least 30% indicated a positive response. No side effects were observed during the test. Plasma Phe concentrations were determined by tandem-mass spectrometry (API 4000 LC/MS/MS System, Shimadzu, Tokyo, Japan).

### Mutation Analysis

Genomic DNA was isolated from peripheral blood samples, 13 exons and related intronic boundaries of the PAH gene were amplified. All PCR products were scanned for mutations by direct sequence analysis, most of which were previously done^32, 33^. All experiments were conducted according to the standard protocol^45, 46^, and the details were described in our previous paper^33^. Mutations were referred to by their description at the DNA level and protein level (http://www.hgvs.org/mutnomen). Since the mutations present in 68 individuals lacked expression data, they were excluded from the analyses that depended on *in vitro* expression information. For mutation classification in relation to BH4-responsiveness, we used the criteria developed by Zurfluh *et al*.^22^, supplemented by the data from BIOPKU database and other published papers^21, 23^.

### Assigning Groups Based on the Assessment of the BH4-Responsiveness-Status of the Detected Mutations

A mutation/allele was regarded as BH4-responsive if it appeared either as homozygous or compound heterozygous form associated with a known null mutation in BH4 responders^22^. BH4-responsiveness was expected in patients with a BH4-responsive mutation on at least one PAH gene copy. We assigned each patient to one of three groups according to the following criteria^47^:

1. “Non-BH4 responsive” group: if both alleles of the patient were null mutations;
2. “BH4 responsive” group: if the patient carried a consistent BH4 responsive mutation on at least one allele;
3. “Undefined BH4 responsive” group: a patient with the presence of at least one mutation encoding a protein with known residual activity but having inconsistent or pending information on BH4 response.

Of 346 patients, 88 patients were assigned to the “BH4 responsive” group, 189 to the “Non-BH4 responsive” group, and the remaining 69 to the “Undefined BH4 responsive” group. We thus attempted to predict the BH4 responsiveness of 346 patients from the respective groups that were assigned based on the information of the single allele.

### Determining the PAH Genotype Severity Using the AV Sum and Classification of BH4 Response

We used an arbitrary assigned value (AV) approach stated by Guldberg *et al*.^34^ to assess the PAH genotype severity of our patients, and the resulting predicted phenotypes from a combination of the two mutant alleles were represented as the sum of the two mutations’ AVs in 278 patients (data were shown in our previous paper)^32^. The ideal cutoff level of AV sum used for predicting the response was determined by the receiver operating characteristic (ROC) curve analysis. Of 278 patients, 88 with an AV sum >2 were classified as “AV sum responders” and 190 with an AV sum = 2 were classified as “AV sum non-responders”.

We investigated the prevalence of BH4 response in those 278 patients who had an AV sum data.

### Assessing the Diagnostic Value of Both Classification Approaches in Predicting BH4 Response

The ability of the AV sum to differentiate the patient’s clinical BH4 response status was validated in 77 BH4-challenged patients having AV estimates for both mutations. Those patients were simultaneously dichotomized into “BH4 responder” and “non-BH4 responder” groups based on their clinical response classification derived from the BH4 loading test and their AV sum. In order to quantify the ability of AV sum to classify BH4 response, sensitivity, specificity, positive predictive value (PPV), and negative predictive value (NPV) were calculated. The ROC curve is a binary tool with five degrees of rating: Excellent (0.9 to 1), good (0.8 to 0.9), fair (0.7 to 0.8), poor (0.6 to 0.7), and not discriminating (0.5 to 0.6)^48^.

The ability of the detected mutations’ BH4 responsiveness-status to discriminate the patient’s clinical BH4 responses status was confirmed in 94 patients loaded with BH4. The patients were designated to one of the three groups (mentioned in Methods: assigning groups based on the assessment of the BH4-responsiveness-status of the detected mutations). The clinical distribution was based on the BH4 loading test. Sensitivity, specificity, PPV, and NPV were calculated to evaluate performance relevant to clinical management.

We used Kappa test to compare the consistency of the two methods (Kappa ≤ 0.20, poor; 0.2 < Kappa ≤ 0.40, fair; 0.4 < Kappa ≤ 0.60, moderate; 0.6  < Kappa ≤ 0.80, good; 0.8 < Kappa, excellent agreement)^49^.

### Statistical Analysis

All statistical analyses were performed using SPSS 17.0 (SPSS Inc., Chicago, Illinois). Initially, all data were analyzed using the Kolmogorov-Smirnov test to assess whether the data were normally distributed. Quantitative data were reported as mean ± standard deviation; normally distributed parameters were compared using the ANOV and non-normally distributed parameters were compared using the Mann-Whitney U test. Qualitative data were compared using the chi-square test; a p-value < 0.05 was considered statistically significant.

## Electronic supplementary material

Supplementary Information
