# Supplementary material for: The Predictive Value of Genetic Analyses in the Diagnosis of Tetrahydrobiopterin (BH4)-Responsiveness in Chinese Phenylalanine Hydroxylase Deficiency Patients
Source: Sci Rep. 2017 Jul 28;7:6762. doi: 10.1038/s41598-017-06462-y (PMC5533732; doi:10.1038/s41598-017-06462-y)
Supplement: Supplementary file 1 — Supplementary Information [file 41598_2017_6462_MOESM1_ESM.doc]

**The predictive value of genetic Analyses in the diagnosis of tetrahydrobiopterin (BH4)-Responsiveness in Chinese Phenylalanine Hydroxylase Deficiency Patients**

Tianwen Zhu^b^, Jun Ye^a^, Lianshu Han^a^, Wenjuan Qiu^a^, Huiwen Zhang^a^, Lili Liang^a^, Xuefan Gu^a^ *

Affiliations:

a Department of Endocrinology and Genetic Metabolism, Xin-Hua Hospital, Shanghai Institute of Pediatric Research Affiliated to Shanghai Jiao Tong University School of Medicine

b Department of Neonatal Medicine, Xin-Hua Hospital Affiliated to Shanghai Jiao Tong University School of Medicine

*Correspondence author at:

Xuefan Gu, M.D. & Ph.D.

Department of Endocrinology and Genetic Metabolism, XinHua Hospital & Shanghai Institute for Pediatric Research Affiliated to Shanghai Jiao Tong University School of Medicine

Kongjiang Road 1665#

Shanghai 200092

China

Tel: +86 21 65011012

Fax +86 21 65791316

E-mail address: gu_xuefan@163.com

**Table S1 Characteristics of 346 Chinese PAH Deficient patients**

| **Patient ID**  M33  M34  M44  M58  M69  M76  M77  M81  M82  M1033  M1049  M1058  M1076  M1079  M1080  M1085  M1102  M1105  M1127  M1140  M1159  M1165  M1229  M1232  M1267  M1268  M1269  M1270  M1275  M1285  M1294  M1300  M1304  M1306  M1307  M1314  M1316  M1317  M1321  M1322  M1323  M1324  M1325  M1334  M1335  M1339  M1340  M1342  M1343  M1346  M1347  M1349  M1360  M1361  M1362  M1374  M1375  M1380  M1384  M1401  M1407  M141  M1412  M1420  M1423  M1437  M1438  M1451  M1463  M1480  M1483  M1490  M1501  M1505  M1514  M1516  M1518  M1536  M1555  M1565  M1574  M1575  M1579  M1590  M1599  M1602  M1604  M1623  M1626  M1630  M164  M1649  M1651  M1660  M1674  M1675  M1686  M1688  M169  M1691  M1697  M1703  M1707  M1709  M1710  M1714  M1721  M1731  M1733  M1742  M1744  M1747  M1753  M1755  M1757  M1767  M1770  M1772  M1773  M1775  M1782  M1783  M1785  M1788  M1799  M1801  M1820  M1823  M1829  M1836  M1837  M1838  M1841  M1845  M1846  M1850  M1851  M1853  M1866  M1867  M1872  M1873  M1881  M1891  M1900  M1903  M1914  M1916  M1919  M1921  M1933  M1941  M1945  M1946  M1950  M1951  M1952  M1963  M1964  M1968  M1970  M1972  M1974  M1980  M1984  M1990  M1992  M1995  M2004  M2005  M2007  M2011  M2016  M2024  M2026  M2028  M2029  M2031  M2041  M2043  M2044  M2049  M2058  M2083  M2086  M2099  M2100  M2101  M2102  M2103  M2108  M2109  M2110  M2111  M2122  M2124  M2125  M2128  M2130  M2131  M2132  M2133  M2136  M2137  M2139  M2141  M2142  M2151  M2158  M2159  M2160  M2161  M2162  M2163  M2165  M2179  M2185  M2206  M2220  M2223  M2225  M2229  M2231  M2234  M2235  M2239  M2241  M2250  M2253  M2254  M2255  M2258  M2267  M2270  M2273  M2277  M2285  M2288  M233  M237  M245  M256  M262  M289  M350  M370  M402  M41  M424  M429  M432  M436  M438  M439  M441  M450  M452  M458  M460  M464  M473  M520  M531  M535  M538  M541  M542  M543  M548  M555  M568  M588  M592  M593  M595  M605  M609  M613  M614  M617  M622  M623  M625  M629  M630  M636  M644  M653  M666  M673  M677  M681  M683  M682  M684  M686  M687  M688  M691  M699  M709  M716  M729  M731  M733  M739  M740  M744  M756  M793  M810  M831  M832  M835  M837  M838  M840  M855  M857  M862  M863  M865  M867  M870  M875  M878  M882  M889  M896  M899  M901  M904  M907  M908  M911  M914  M915  M917  M920  M922  M936  M960  M952  M974  M987  M996 | **Gender**  F  M  M  M  F  M  F  F  M  M  M  M  M  M  F  M  M  F  F  F  F  F  M  F  M  F  F  M  M  M  F  F  F  F  M  F  F  F  F  M  M  F  M  M  F  F  F  F  F  F  M  M  F  F  F  F  F  F  M  F  F  M  M  F  M  M  M  M  F  M  F  F  M  M  F  F  F  F  M  F  F  F  F  M  F  F  M  M  M  F  M  M  F  F  M  F  F  M  M  F  F  F  F  F  F  F  F  M  F  M  F  F  F  F  F  F  M  F  M  M  M  F  F  F  M  F  M  M  M  F  M  F  F  F  F  F  M  F  F  M  M  M  M  F  F  M  M  F  F  M  F  F  F  F  F  F  M  F  F  M  M  F  M  M  M  M  F  F  F  M  M  F  M  M  F  M  M  F  M  M  F  F  F  F  M  F  M  F  M  F  M  F  M  M  M  F  F  M  M  M  M  F  F  F  F  F  M  M  M  M  M  F  M  M  M  M  M  M  F  F  F  M  F  F  F  M  F  M  M  M  M  M  M  M  F  F  M  M  M  M  F  F  M  M  M  F  F  F  F  F  M  F  F  F  M  M  M  F  M  M  M  F  F  M  F  F  M  F  F  M  M  F  F  F  M  F  F  F  F  M  F  M  M  M  M  M  M  M  M  F  F  M  M  M  M  M  F  F  F  M  M  F  M  F  M  M  F  F  M  M  F  F  M  M  F  M  M  M  M  F  F  M  M  M  M  M  M  M  M  F  M  F  M  F  M  M  M  F  F  M  F  F  M  F  F  M | **Pre-Phe**  1560  1680  1080  1202  1680  2160  456  1080  1920  820.8  1000.8  823.8  1260  936  660  469.8  660  616.8  258  426.6  1260  1020  607.2  896.4  1593  1830  2057.4  1788  1500  835.8  627.6  600.6  1500  1201  1428  588  876  780  1560  600  759  1260  2100  1080  1320  1920  1080  726.6  562.2  664.8  970.2  1218  900  876  451.8  660  1020  990  841.8  564  1020  2532  1260  1260  1080  1020  1320  489  510  720  1260  354.6  1320  960  1378.2  1020  1296  1869  1525.8  1680  1104  2400  714  1373.4  1320  600  959.4  1108.2  1320  1920  540  900  1500  816  500.4  510  420  198  1020  792  1699.8  1014  360  384  931.2  1506  372  408  1071  1080  1440  1504.8  694.2  1115.4  1116  1326  1651.8  228  960  1335  1218  1734  1980  820.2  1141.2  640.8  666  1240  880.8  1110  924  1837.8  1380  1260  1800  189  1012.2  822  1560  257.4  2400  1500  1380  1260  609.6  1002  1254  1320  1638  559.2  660  2160  2280  780  342  1242  624  480  480  540  1248  2460  1320  1290  2937  1600.2  495.6  1760.4  900  594  1225.8  720  1125.6  720  1558.2  132  1002  212.4  1380  1284.6  1671  608.4  773.4  834  1208  720  2100  876  981.6  2208  1020  1374  132.42  1020  1080  1284  904.8  2880  2082  771  1086  720  1250  1591.8  2100  1140  1230  660  318  1757.4  1668  1260  1560  1500  666.6  502.8  1599.6  1386  1800  1183.2  1050  1572  1980  169.2  1308  720  2160  1800  342  480  1260  432.6  1980  762  660  720  151.8  1680  1320  1920  960  1326  2100  1260  1270  960  942  1260  720  600  1320  1560  1440  2340  1080  1205  960  1560  1214  600  1226  1512  1290  1228  1230  1240  1210  720  1500  1202  1740  1860  1216  1380  1320  2340  2160  1440  1320  1209  1300  1092  1380  1620  1500  1380  960  1680  960  1800  600  1320  1980  1260  1200  1620  1560  1740  1230  1620  1560  1548  1440  2100  1740  2400  1860  2100  1215  1740  849  1140  1304.4  833.4  1140  540  780  780  938.4  360  1080  900  900  330  720  960  804  660  660  2040  960  534  1740  660  1734  249  1680  2400  1006.2  627  1920  1260  576  1233.6  552  894 | **Phenotype**  cPKU  cPKU  mPKU  cPKU  cPKU  cPKU  mPKU  mPKU  cPKU  mPKU  mPKU  mPKU  cPKU  mPKU  mPKU  mPKU  mPKU  mPKU  MHP  mPKU  cPKU  mPKU  mPKU  mPKU  cPKU  cPKU  cPKU  cPKU  cPKU  mPKU  mPKU  mPKU  cPKU  cPKU  cPKU  mPKU  mPKU  mPKU  cPKU  mPKU  mPKU  cPKU  cPKU  mPKU  cPKU  cPKU  mPKU  mPKU  mPKU  mPKU  mPKU  cPKU  mPKU  mPKU  mPKU  mPKU  mPKU  mPKU  mPKU  mPKU  mPKU  cPKU  cPKU  cPKU  mPKU  mPKU  cPKU  mPKU  mPKU  mPKU  cPKU  MHP  cPKU  mPKU  cPKU  mPKU  cPKU  cPKU  cPKU  cPKU  mPKU  cPKU  mPKU  cPKU  cPKU  mPKU  mPKU  mPKU  cPKU  cPKU  mPKU  mPKU  cPKU  mPKU  mPKU  mPKU  mPKU  MHP  mPKU  mPKU  cPKU  mPKU  MHP  mPKU  mPKU  cPKU  mPKU  mPKU  mPKU  mPKU  cPKU  cPKU  mPKU  mPKU  mPKU  cPKU  cPKU  MHP  mPKU  cPKU  cPKU  cPKU  cPKU  mPKU  mPKU  mPKU  mPKU  cPKU  mPKU  mPKU  mPKU  cPKU  cPKU  cPKU  cPKU  MHP  mPKU  mPKU  cPKU  MHP  cPKU  cPKU  cPKU  cPKU  mPKU  mPKU  cPKU  cPKU  cPKU  mPKU  mPKU  cPKU  cPKU  mPKU  MHP  cPKU  mPKU  mPKU  mPKU  mPKU  cPKU  cPKU  cPKU  cPKU  cPKU  cPKU  mPKU  cPKU  mPKU  mPKU  cPKU  mPKU  mPKU  mPKU  cPKU  MHP  mPKU  MHP  cPKU  cPKU  cPKU  mPKU  mPKU  mPKU  cPKU  mPKU  cPKU  mPKU  mPKU  cPKU  mPKU  cPKU  MHP  mPKU  mPKU  cPKU  mPKU  cPKU  cPKU  mPKU  mPKU  mPKU  cPKU  cPKU  cPKU  mPKU  cPKU  mPKU  MHP  cPKU  cPKU  cPKU  cPKU  cPKU  mPKU  mPKU  cPKU  cPKU  cPKU  mPKU  mPKU  cPKU  cPKU  MHP  cPKU  mPKU  cPKU  cPKU  MHP  mPKU  cPKU  mPKU  cPKU  mPKU  mPKU  mPKU  MHP  cPKU  cPKU  cPKU  mPKU  cPKU  cPKU  cPKU  cPKU  mPKU  mPKU  cPKU  mPKU  mPKU  cPKU  cPKU  cPKU  cPKU  mPKU  cPKU  mPKU  cPKU  cPKU  mPKU  cPKU  cPKU  cPKU  cPKU  cPKU  cPKU  cPKU  mPKU  cPKU  cPKU  cPKU  cPKU  cPKU  cPKU  cPKU  cPKU  cPKU  cPKU  cPKU  cPKU  cPKU  mPKU  cPKU  cPKU  cPKU  cPKU  mPKU  cPKU  mPKU  cPKU  mPKU  cPKU  cPKU  cPKU  cPKU  cPKU  cPKU  cPKU  cPKU  cPKU  cPKU  cPKU  cPKU  cPKU  cPKU  cPKU  cPKU  cPKU  cPKU  cPKU  mPKU  mPKU  cPKU  mPKU  mPKU  mPKU  mPKU  mPKU  mPKU  MHP  mPKU  mPKU  mPKU  MHP  mPKU  mPKU  mPKU  mPKU  mPKU  cPKU  mPKU  mPKU  cPKU  mPKU  cPKU  MHP  cPKU  cPKU  mPKU  mPKU  cPKU  cPKU  mPKU  cPKU  mPKU  mPKU | **Genotype**  c.[728G>A];[728G>A]  c.[722delG];[1199G>A]  c.[510-2A>G];[1199G>C]  c.[770G>T];[1197A>T]  c.[442-1G>A];[1068C>A]  c.[728G>A];[728G>A]  c.[722G>A];[1139C>T]  c.[965C>A];[1242C>A]  c.[208_210delTCT];[611A>G]  c.[728G>A];[842+2T>A]  c.[442-1G>A];[611A>G]  c.[728G>A];[1238G>C]  c.[331C>T];[1238G>C]  c.[724C>T];[1197A>T]  c.[442-1G>A];[721C>T]  c.[721C>T];[1162G>A]  c.[611A>G];[728G>A]  c.[721C>T];[728G>A]  c.[721C>T];[721C>T]  c.[331C>T];[1197A>T]  c.[460T>C];[977G>A]  c.[728G>A];[728G>A]  c.[721C>T];[842+2T>A]  c.[611A>G];[728G>A]  c.[331C>T];[1068C>A]  c.[728G>A];[1071C>A]  c.[331C>T];[1238G>C]  c.[707-1G>A];[722delG]  c.[728G>A];[728G>A]  c.[1197A>T];[1238G>C]  c.[721C>T];[842+1G>A]  c.[611A>G];[721C>T]  c.[498C>A];[611A>G]  c.[611A>G]; [728G>A]  c.[1068C>A];[1238G>C]  c.[611A>G];[721C>T]  c.[611A>G];[728G>A]  c.[442-1G>A];[721C>T]  c.[728G>A];[728G>A]  c.[680T>A];[728G>A]  c.[611A>G];[724C>T]  c.[158G>A];[1222C>T]  c.[707-1G>A];[728G>A]  c.[728G>A];[740G>T]  c.[728G>A];[1238G>C]  c.[1238G>C];[1238G>C]  c.[728G>A];[1301C>A]  c.[740G>T];[1070G>A]  c.[721C>T];[764T>C]  c.[611A>G];[770G>T]  c.[611A>G];[754C>T]  c.[331C>T];[1197A>T]  c.[442-1G>A];[721C>T]  c.[611A>G];[728G>A]  c.[482T>C];[721C>T]  c.[721C>T];[1199G>A]  c.[611A>G];[721C>T]  c.[194T>C];[682G>T]  c.[208T>C];[1070G>A]  c.[611A>G];[611A>G]  c.[611A>G];[1100T>G]  c.[331C>T];[728G>A]  c.[611A>G];[781C>T]  c.[526C>T];[707-1G>A]  c.[721C>T];[728G>A]  c.[823C>G];[1068C>A]  c.[442-1G>A];[1238G>C]  c.[331C>T];[721C>T]  c.[331C>T];[1197A>T]  c.[728G>A];[782G>A]  c.[728G>A];[1197A>T]  c.[721C>T];[728G>A]  c.[728G>A];[1315+4A>G]  c.[611A>G];[721C>T]  c.[728G>A];[728G>A]  c.[482T>C];[728G>A]  c.[331C>T];[827T>G]  c.[728G>A];[727C>T]  c.[611A>G];[1068C>A]  c.[728G>A];[728G>A]  c.[331C>T];[728G>A]  c.[331C>T];[728G>A]  c.[1162G>A];[1197A>T]  c.[611A>G];[728G>A]  c.[611A>G];[728G>A]  c.[728G>A];[1301C>A]  c.[721C>T];[728G>A]  c.[728G>A];[728G>A]  c.[510T>A];[838G>A]  c.[728G>A];[1068C>A]  c.[728G>A];[1223G>A]  c.[781C>T];[1068C>A]  c.[728G>A];[1301C>A]  c.[728G>A];[728G>A]  c.[1068C>A];[1301C>A]  c.[331C>T];[721C>T]  c.[721C>T];[728G>A]  c.[728G>A];[1256A>G]  c.[728G>A];[1068C>A]  c.[331C>T];[611A>G]  c.[611A>G];[611A>G]  c.[526C>T];[1162G>A]  c.[721C>T];[728G>A]  c.[611A>G];[1256A>G]  c.[611A>G];[1238G>C]  c.[331C>T];[1068C>A]  c.[194T>C];[722G>A]  c.[208_210delTCT];[721C>T]  c.[728G>A];[1197A>T]  c.[611A>G];[1197A>T]  c.[611A>G];[728G>A]  c.[694C>T];[728G>A]  c.[611A>G];[1068C>A]  c.[611A>G];[728G>A]  c.[611A>G];[611A>G]  c.[331C>T];[1068C>A]  c.[442-1G>A];[1217T>C]  c.[464G>A];[1197A>T]  c.[442-1G>A];[1197A>T]  c.[728G>A];[728G>A]  c.[442-1G>A];[728G>A]  c.[1068C>A];[1197A>T]  c.[611A>G];[1162G>A]  c.[1222C>T];[1238G>C]  c.[331C>T];[1068C>A]  c.[442-1G>A];[1301C>A]  c.[728G>A];[755G>A]  c.[208_210delTCT];[194T>C]  c.[482T>C];[1197A>T]  c.[331C>T];[442-1G>A]  c.[611A>G];[721C>T]  c.[442-1G>A];[1301C>A]  c.[728G>A];[728G>A]  c.[331C>T];[755G>A]  c.[611A>G];[728G>A]  c.[611A>G];[1174T>A]  c.[728G>A];[1197A>T]  c.[208_210delTCT];[473G>A]  c.[331C>T];[611A>G]  c.[464G>A];[728G>A]  c.[470G>T];[728G>A]  c.[611A>G];[728G>A]  c.[728G>A];[782G>A]  c.[611A>G];[728G>A]  c.[728G>A];[1200G>T]  c.[728G>A];[775G>A]  c.[442-1G>A];[782G>A]  c.[190_194delCACAT];[466G>C]  c.[1154T>C];[1154T>C]  c.[721C>T];[728G>A]  c.[728G>A];[728G>A]  c.[165T>G];[1024delG]  c.[611A>G];[1197A>T]  c.[721C>T];[728G>A]  c.[611A>G];[721C>T]  c.[611A>G];[728G>A]  c.[331C>T];[728G>A]  c.[728G>A];[1223G>A]  c.[331C>T];[721C>T]  c.[498C>A];[721C>T]  c.[560G>A];[929C>T]  c.[728G>A];[728G>A]  c.[331C>T];[728G>A]  c.[728G>A];[728G>A]  c.[1068C>A];[1197A>T]  c.[728G>A];[793T>C]  c.[721C>T];[728G>A]  c.[442-1G>A];[728G>A]  c.[611A>G];[541-543delGAG]  c.[721C>T];[728G>A]  c.[331C>T];[611A>G]  c.[721C>T];[1197A>T]  c.[194T>C];[728G>A]  c.[721C>T];[728G>A]  c.[331C>T];[611A>G]  c.[184C>G];[1238G>C]  c.[728G>A];[1033G>A]  c.[320A>G];[1223G>A]  c.[1238G>C];[1238G>C]  c.[728G>A];[1068C>A]  c.[611A>G];[611A>G]  c.[739G>C];[1238G>C]  c.[728G>A];[1199G>C]  c.[721C>T];[1238G>C]  c.[728G>A];[1197A>T]  c.[540-544delGGAGG];[1172G>T]  c.[722delG];[728G>A]  c.[442-1G>A];[721C>T]  c.[707-1G>A];[1223G>A]  c.[1068C>A];[1066-14C>G]  c.[331C>T];[1301C>A]  c.[442-1G>A];[728G>A]  c.[728G>A];[1123C>G]  c.[208_210delTCT];[728G>A]  c.[442-1G>A];[611A>G]  c.[728G>A];[1238G>C]  c.[611A>G];[721C>T]  c.[611A>G];[842+2T>A]  c.[331C>T];[728G>A]  c.[728G>A];[1238G>C]  c.[158G>A];[842+2T>A]  c.[442-1G>A];[721C>T]  c.[728G>A];[1199G>C]  c.[442-1G>A];[611A>G]  c.[1238G>C];[1238G>C]  c.[728G>A];[728G>A]  c.[721C>T];[728G>A]  c.[331C>T];[842+5G>A]  c.[442-1G>A];[1243G>A]  c.[208_210delTCT];[722delG]  c.[331C>T];[1250A>G]  c.[722delG];[728G>A]  c.[728G>A];[755G>A]  c.[1068C>A];[1238G>C]  c.[228G>C];[470G>A]  c.[728G>A];[1315+6T>A]  c.[331C>T];[728G>A]  c.[901C>T];[1315+4A>G]  c.[442-1G>A];[782G>A]  c.[611A>G];[728G>A]  c.[728G>A];[842+2T>A]  c.[611A>G];[1197A>T]  c.[1068C>A];[1197A>T]  c.[158G>A];[728G>A]  c.[611A>G];[707-1G>A]  c.[611A>G];[722G>A]  c.[441+3G>C];[611A>G]  c.[1068C>A];[1197A>T]  c.[611A>G];[1243G>A]  c.[721C>T];[833C>T]  c.[331C>T];[728G>A]  c.[721C>T];[728G>A]  c.[331C>T];[1238G>C]  c.[466G>C];[1223G>A]  c.[472C>T];[1068C>A]  c.[721C>T];[1043T>C]  c.[721C>T];[1315+6T>A]  c.[728G>A];[728G>A]  c.[728G>A];[1238G>C]  c.[1068C>A];[1197A>T]  c.[194T>C];[611A>G]  c.[728G>A];[728G>A]  c.[611A>G];[707-1G>A]  c.[1068C>A];[1238G>C]  c.[441+3G>C];[611A>G]  c.[728G>A];[1162G>A]  c.[728G>A];[1162G>A]  c.[728G>A];[728G>A]  c.[728G>A];[1197A>T]  c.[441+3G>C];[1197A>T]  c.[1068C>A];[1197A>T]  c.[838G>A];[1068C>A]  c.[113_115delTCT];[716G>A]  c.[442-1G>A];[728G>A]  c.[442-1G>A];[1068C>A]  c.[728G>A];[929C>T]  c.[472C>T];[1199G>C]  c.[611A>G];[611A>G]  c.[977G>A];[1197A>T]  c.[442-1G>A];[707-1G>A]  c.[331C>T];[842+2T>A]  c.[728G>A];[728G>A]  c.[671T>C];[764T>C]  c.[461A>G];[728G>A]  c.[728G>A];[728G>A]  c.[611A>G];[617A>G]  c.[728G>A];[728G>A]  c.[331C>T];[728G>A]  c.[722delG];[728G>A]  c.[728G>A];[1068C>A]  c.[498C>G];[728G>A]  c.[442-1G>A];[728G>A]  c.[168G>T];[728G>A]  c.[331C>T];[611A>G]  c.[466G>C];[728G>A]  c.[442-1G>A];[1068C>A]  c.[509+1G>A];[728G>A]  c.[1024G>A];[1238G>C]  c.[526C>T];[728G>A]  c.[770G>T];[707-1G>A]  c.[707-1G>A];[1238G>C]  c.[1024G>A];[1238G>C]  c.[331C>T];[1238G>C]  c.[442-1G>A];[728G>A]  c.[728G>A];[1085C>T]  c.[1197A>T];[1199G>A]  c.[442-1G>A];[1197A>T]  c.[728G>A];[977G>A]  c.[514C>T];[1033G>A];  c.[611A>G];[728G>A]  c.[331C>T];[498C>A]  c.[441+2T>A];[1068C>A]  c.[331C>T];[1068C>A]  c.[[611A>G](http://www.biopku.org/pah/result-details-pah.asp?ID=818)];[728G>A]  c.[442-1G>A];[[722delG](http://www.biopku.org/pah/result-details-pah.asp?ID=212)]  [c.[722delG](http://www.biopku.org/pah/result-details-pah.asp?ID=212)];[728G>A]  c.[728G>A];[1197A>T]  c.[208_210delTCT];[442-1G>A]  c.[442-1G>A];[728G>A]  c.[770G>T];[1068C>A]  c.[728G>A];[1199G>C]  c.[722delG];[728G>A]  c.[1049C>A];[1066-14C>G]  c.[442-1G>A];[728G>A]  c.[728G>A];[1068C>A]  c.[168+5G>C];[442-1G>A]  c.[770G>T];[770G>T]  c.[728G>A];[728G>A]  c.[611A>G];[1232C>G]  c.[611A>G];[728G>A]  c.[721C>T];[1238G>C]  c.[707-1G>A];[611A>G]  c.[724C>T];[1162G>A]  c.[721C>T];[842+2T>A]  c.[470G>A];[611A>G]  c.[1045T>G];[721C>T]  c.[208_210delTCT];[1162G>A]  c. [728G>A];[971T>A]  c.[728G>A];[1162G>A]  c.[611A>G];[721C>T]  c.[728G>A];[1238G>C]  c.[442-1G>A];[721C>T]  c.[770G>T];[1197A>T]  c.[208_210delTCT];[782G>A]  c.[498C>A];[498C>A]  c.[208_210delTCT];[728G>A]  c.[548A>G];[721C>T]  c.[810A>T];[1223G>A]  c.[721C>T];[856G>A]  c.[611A>G];[707-1G>A]  c.[728G>A];[728G>A]  c.[361T>C]];[721C>T]  c.[611A>G];[728G>A]  c.[838G>A];[1301C>A]  c.[472C>T];[1197A>T]  c.[721C>T];[739G>C]  c.[442-1G>A];[1068C>G]  c.[442-1G>A];[1238G>C]  c.[770G>T];[1223G>A]  c.[728G>A];[827T>A]  c.[442-1G>A];[1238G>C]  c.[611A>G];[728G>A]  c.[721C>T];[856G>A]  c.[331C>T];[1197A>T]  c.[721C>T];[739G>C]  c.[466G>C];[1223G>A] | **Protein variation**  p.[Arg243Gln];[Arg243Gln]  p.[Arg241Profs*100];[Arg400Lys]  p.[IVS5-2A>G];[Arg400Thr]  p.[Gly257Val];[Val399Val]  p.[IVS4-1G>A];[Tyr356*]  p.[Arg243Gln];[Arg243Gln]  p.[Arg241His];[Thr380Met]  p.[Ala322Asp];[Tyr414*]  p.[Ser70del];[Ex6-96A>G]  p.[Arg243Gln];[IVS7+2T>A]  p.[IVS4-1G>A];[Ex6-96A>G]  p.[Arg243Gln];[Arg413Pro]  p.[Arg111*];[Arg413Pro]  p.[Leu242Phe];[Val399Val]  p.[IVS4-1G>A];[Arg241Cys]  p.[Arg241Cys];[Val388Met]  p.[Ex6-96A>G];[Arg243Gln]  p.[Arg241Cys];[Arg243Gln]  p.[Arg241Cys];[Arg241Cys]  p.[Arg111*];[Val399Val]  p.[Tyr154His];[Trp326*]  p.[Arg243Gln];[Arg243Gln]  p.[Arg241Cys];[IVS7+2T>A]  p.[Ex6-96A>G];[Arg243Gln]  p.[Arg111*];[Tyr356*]  p.[Arg243Gln];[Cys357*]  p.[Arg111*];[Arg413Pro]  p.[IVS6-1G>A];[Arg241Profs*100]  p.[Arg243Gln];[Arg243Gln]  p.[Val399Val];[Arg413Pro]  p.[Arg241Cys];[IVS7+1G>A]  p.[Ex6-96A>G]; [Arg241Cys]  p.[Tyr166*];[Ex6-96A>G]  p.[Ex6-96A>G]; [Arg243Gln]  p.[Tyr356*];[Arg413Pro]  p.[Ex6-96A>G];[Arg241Cys]  p.[Ex6-96A>G];[Arg243Gln]  p. [IVS4-1G>A];[Arg241Cys]  p.[Arg243Gln];[Arg243Gln]  p.[Leu227Gln];[Arg243Gln]  p.[Ex6-96A>G];[Leu242Phe]  p.[Arg53His];[Arg408Trp]  p.[IVS6-1G>A];[Arg243Gln]  p.[Arg243Gln];[Gly247Val]  p.[Arg243Gln];[Arg413Pro]  p.[Arg413Pro];[Arg413Pro]  p.[Arg243Gln];[Ala434Asp]  p.[Gly247Val];[Cys357Tyr]  p.[Arg241Cys];[Leu255Ser]  p.[Ex6-96A>G];[Gly257Val]  p.[Ex6-96A>G];[Arg252Trp]  p.[Arg111*];[Val399Val]  p.[IVS4-1G>A];[Arg241Cys]  p.[Ex6-96A>G];[Arg243Gln]  p.[Phe161Ser];[Arg241Cys]  p.[Arg241Cys];[Arg400Lys]  p.[Ex6-96A>G];[Arg241Cys]  p.[Ile65Thr];[Glu228*]  p.[Ser70Pro];[Cys357Tyr]  p.[Ex6-96A>G];[Ex6-96A>G]  p.[Ex6-96A>G];[Leu367Arg]  p.[Arg111*];[Arg243Gln]  p.[Ex6-96A>G];[Arg261*]  p.[Arg176*];[IVS6-1G>A]  p.[Arg241Cys];[Arg243Gln]  p.[Pro275Ala];[Tyr356*]  p.[IVS4-1G>A];[Arg413Pro]  p.[Arg111*];[Arg241Cys]  p.[Arg111*];[Val399Val]  p.[Arg243Gln];[Arg261Gln]  p.[Arg243Gln];[Val399Val]  p.[Arg241Cys];[Arg243Gln]  p.[Arg243Gln];[IVS12+4A>G]  p.[Ex6-96A>G];[Arg241Cys]  p.[Arg243Gln];[Arg243Gln]  p.[Phe161Ser];[Arg243Gln]  p.[Arg111*];[Met276Arg]  p.[Arg243Gln];[Arg243*]  p.[Ex6-96A>G];[Tyr356*]  p.[Arg243Gln];[Arg243Gln]  p.[Arg111*];[Arg243Gln]  p.[Arg111*];[Arg243Gln]  p.[Val388Met];[Val399Val]  p.[Ex6-96A>G];[Arg243Gln]  p.[Ex6-96A>G];[Arg243Gln]  p.[Arg243Gln];[Ala434Asp]  p.[Arg241Cys];[Arg243Gln]  p.[Arg243Gln];[Arg243Gln]  p.[His170Gln];[Glu280Gln]  p.[Arg243Gln];[Tyr356*]  p.[Arg243Gln];[Arg408Gln]  p.[Arg261*];[Tyr356*]  p.[Arg243Gln];[Ala434Asp]  p.[Arg243Gln];[Arg243Gln]  p.[Tyr356*];[Ala434Asp]  p.[Arg111*];[Arg241Cys]  p.[Arg241Cys];[Arg243Gln]  p.[Arg243Gln];[Gln419Arg]  p.[Arg243Gln];[Tyr356*]  p.[Arg111*];[Ex6-96A>G]  p.[Ex6-96A>G];[Ex6-96A>G]  p.[Arg176*];[Val388Met]  p.[Arg241Cys];[Arg243Gln]  p.[Ex6-96A>G];[Gln419Arg]  p.[Ex6-96A>G];[Arg413Pro]  p.[Arg111*];[Tyr356*]  p.[Ile65Thr];[Arg241His]  p.[Ser70del];[Arg241Cys]  p.[Arg243Gln];[Val399Val]  p.[Ex6-96A>G];[Val399Val]  p.[Ex6-96A>G];[Arg243Gln]  p.[Gln232*];[Arg243Gln]  p.[Ex6-96A>G];[Tyr356*]  p.[Ex6-96A>G];[Arg243Gln]  p.[Ex6-96A>G];[Ex6-96A>G]  p.[Arg111*];[Tyr356*]  p.[IVS4-1G>A];[Ile406Thr]  p.[Arg155His];[Val399Val]  p.[IVS4-1G>A];[Val399Val]  p.[Arg243Gln];[Arg243Gln]  p.[IVS4-1G>A];[Arg243Gln]  p.[Tyr356*];[Val399Val]  p.[Ex6-96A>G];[Val388Met]  p.[Arg408Trp];[Arg413Pro]  p.[Arg111*];[Tyr356*]  p.[IVS4-1G>A];[Ala434Asp]  p.[Arg243Gln];[Arg252Gln]  p.[Ser70del];[Ile65Thr]  p.[Phe161Ser];[Val399Val]  p.[Arg111*];[IVS4-1G>A]  p.[Ex6-96A>G];[Arg241Cys]  p.[IVS4-1G>A];[Ala434Asp]  p.[Arg243Gln];[Arg243Gln]  p.[Arg111*];[Arg252Gln]  p.[Ex6-96A>G];[Arg243Gln]  p.[Ex6-96A>G];[Phe392Ile]  p.[Arg243Gln];[Val399Val]  p.[Ser70del];[Arg158Gln]  p.[Arg111*];[Ex6-96A>G]  p.[Arg155His];[Arg243Gln]  p.[Arg157Ile];[Arg243Gln]  p.[Ex6-96A>G];[Arg243Gln]  p.[Arg243Gln];[Arg261Gln]  p.[Ex6-96A>G];[Arg243Gln]  p.[Arg243Gln];[Arg400Ser]  p.[Arg243Gln];[Ala259Thr]  p.[IVS4-1G>A];[Arg261Gln]  p.[His64*];[Ala156Pro]  p.[Leu385Pro];[Leu385Pro]  p.[Arg241Cys];[Arg243Gln]  p.[Arg243Gln];[Arg243Gln]  p.[Phe55Leu];[Ala342Hisfs*58]  p.[Ex6-96A>G];[Val399Val]  p.[Arg241Cys];[Arg243Gln]  p.[Ex6-96A>G];[Arg241Cys]  p.[Ex6-96A>G];[Arg243Gln]  p.[Arg111*];[Arg243Gln]  p.[Arg243Gln];[Arg408Gln]  p.[Arg111*];[Arg241Cys]  p.[ Tyr166*];[Arg241Cys]  p.[Trp187*];[Ser310Phe]  p.[Arg243Gln];[Arg243Gln]  p.[Arg111*];[Arg243Gln]  p.[Arg243Gln];[Arg243Gln]  p.[ Tyr356*];[Val399Val]  p.[Arg243Gln];[Cys265Arg)]  p.[Arg241Cys];[Arg243Gln]  p.[IVS4-1G>A];[Arg243Gln]  p.[Ex6-96A>G];[181Gludel]  p.[Arg241Cys];[Arg243Gln]  p.[Arg111*];[Ex6-96A>G]  p.[Arg241Cys];[Val399Val]  p.[Ile65Thr];[Arg243Gln]  p.[Arg241Cys];[Arg243Gln]  p.[Arg111*];[Ex6-96A>G]  p.[Leu62Val];[Arg413Pro]  p.[Arg243Gln];[Ala345Thr]  p.[His107Arg];[Arg408Gln]  p.[Arg413Pro];[Arg413Pro]  p.[Arg243Gln];[Tyr356*]  p.[Ex6-96A>G];[Ex6-96A>G]  p.[Gly247Arg];[Arg413Pro]  p.[Arg243Gln];[Arg400Thr]  p.[Arg241Cys];[Arg413Pro]  p.[Arg243Gln];[Val399Val]  p.[Met180*];[Ser391Ile]  p.[Arg241Profs*100];[Arg243Gln]  p.[IVS4-1G>A];[Arg241Cys]  p.[IVS6-1G>A];[Arg408Gln]  p.[Tyr356*];[IVS10-14C>G]  p.[Arg111*];[Ala434Asp]  p.[IVS4-1G>A];[Arg243Gln]  p.[Arg243Gln];[Gln375Glu]  p.[Ser70del];[Arg243Gln]  p.[IVS4-1G>A];[Ex6-96A>G]  p.[Arg243Gln];[Arg413Pro]  p.[Ex6-96A>G];[Arg241Cys]  p.[Ex6-96A>G];[IVS7+2T>A]  p.[Arg111*];[Arg243Gln]  p.[Arg243Gln];[Arg413Pro]  p.[Arg53His];[IVS7+2T>A]  p.[IVS4-1G>A];[Arg241Cys]  p.[Arg243Gln];[Arg400Thr]  p.[IVS4-1G>A];[Ex6-96A>G]  p.[Arg413Pro];[Arg413Pro]  p.[Arg243Gln];[Arg243Gln]  p.[Arg241Cys];[Arg243Gln]  p.[Arg111*];[IVS7+5G>A]  p.[IVS4-1G>A];[Asp415Asn]  p.[Ser70del];[Arg241Profs*100]  p.[Arg111*];[Tyr417Cys]  p.[Arg241Profs*100];[Arg243Gln]  p.[Arg243Gln];[Arg252Gln]  p.[ Tyr356*];[Arg413Pro]  p.[Glu76Asp];[Arg157Lys]  p.[Arg243Gln];[IVS12+6T>A]  p.[Arg111*];[Arg243Gln]  p.[Gln301*];[IVS12+4A>G]  p.[IVS4-1G>A];[Arg261Gln]  p.[Ex6-96A>G];[Arg243Gln]  p.[Arg243Gln];[IVS7+2T>A]  p.[Ex6-96A>G];[Val399Val]  p.[ Tyr356*];[Val399Val]  p.[Arg53His];[Arg243Gln]  p.[Ex6-96A>G];[IVS6-1G>A]  p.[Ex6-96A>G];[Arg241His]  p.[IVS4+3G>C];[Ex6-96A>G]  p.[Tyr356*];[Val399Val]  p.[Ex6-96A>G];[Asp415Asn]  p.[Arg241Cys];[Thr278Ile]  p.[Arg111*];[Arg243Gln]  p.[Arg241Cys];[Arg243Gln]  p.[Arg111*];[Arg413Pro]  p.[Ala156Pro];[Arg408Gln]  p.[Arg158Trp];[Tyr356*]  p.[Arg241Cys];[Leu348Pro]  p.[Arg241Cys];[IVS12+6T>A]  p.[Arg243Gln];[Arg243Gln]  p.[Arg243Gln];[Arg413Pro]  p.[Tyr356*];[Val399Val]  p.[Ile65Thr];[Ex6-96A>G]  p.[Arg243Gln];[Arg243Gln]  p.[Ex6-96A>G];[IVS6-1G>A]  p.[Tyr356*];[Arg413Pro]  p.[IVS4+3G>C];[Ex6-96A>G]  p.[Arg243Gln];[Val388Met]  p.[Arg243Gln];[Val388Met]  p.[Arg243Gln];[Arg243Gln]  p.[Arg243Gln];[Val399Val]  p.[IVS4+3G>C];[Val399Val]  p.[Tyr356*];[Val399Val]  p.[Glu280Lys];[Tyr356*]  p.[Phe39del];[Gly239Asp]  p.[IVS4-1G>A];[Arg243Gln]  p.[IVS4-1G>A];[Tyr356*]  p.[Arg243Gln];[Ser310Phe)]  p.[Arg158Trp];[Arg400Thr]  p.[Ex6-96A>G];[Ex6-96A>G]  p.[Trp326*];[Val399Val]  p.[IVS4-1G>A];[IVS6-1G>A]  p.[Arg111*];[IVS7+2T>A]  p.[Arg243Gln];[Arg243Gln]  p.[Ile224Thr];[Leu255Ser]  p.[Tyr154Cys];[Arg243Gln]  p.[Arg243Gln];[Arg243Gln]  p.[Ex6-96A>G];[Tyr206Cys]  p.[Arg243Gln];[Arg243Gln]  p.[Arg111*];[Arg243Gln]  p.[Arg241Profs*100];[Arg243Gln]  p.[Arg243Gln];[Tyr356*]  p.[Tyr166*];[Arg243Gln]  p.[IVS4-1G>A];[Arg243Gln]  p.[Glu56Asp];[Arg243Gln]  p.[Arg111*];[Ex6-96A>G]  p.[Ala156Pro];[Arg243Gln]  p.[IVS4-1G>A];[Tyr356*]  p.[IVS5+1G>A];[Arg243Gln]  p.[Ala342Thr];[Arg413Pro]  p.[Arg176*];[Arg243Gln]  p.[Gly257Val];[IVS6-1G>A]  p.[IVS6-1G>A];[Arg413Pro]  p.[Ala342Thr];[Arg413Pro]  p.[Arg111*];[Arg413Pro]  p.[IVS4-1G>A];[Arg243Gln]  p.[Arg243Gln];[Pro362Leu]  p.[Val399Val];[Arg400Lys]  p.[IVS4-1G>A];[Val399Val]  p.[Arg243Gln];[Trp326*]  p.[Gln172*];[Ala345Thr]  p.[Ex6-96A>G];[Arg243Gln]  [p.[Arg111*](http://www.biopku.org/pah/result-details-pah.asp?ID=11)];[Tyr166*]  p.[IVS4+2T>A];[Tyr356*]  [p.[Arg111*](http://www.biopku.org/pah/result-details-pah.asp?ID=11)];[Tyr356*]  p.[Ex6-96A>G];[Arg243Gln]  p.[IVS4-1G>A];[Arg241Profs*100]  p.[Arg241Profs*100];[Arg243Gln]  p.[Arg243Gln];[Val399Val]  p.[Ser70del];[IVS4-1G>A]  p.[IVS4-1G>A];[Arg243Gln]  p.[Gly257Val];[Tyr356*]  p.[Arg243Gln];[Arg400Thr]  p.[Arg241Profs*100];[Arg243Gln]  p.[Ser350Tyr];[IVS10-14C>G]  p.[IVS4-1G>A];[Arg243Gln]  p.[Arg243Gln];[Tyr356*]  p.[IVS2+5G>C];[IVS4-1G>A]  p.[Gly257Val];[Gly257Val]  p.[Arg243Gln];[Arg243Gln]  p.[Ex6-96A>G];[Ser411*]  p.[Ex6-96A>G];[Arg243Gln]  p.[Arg241Cys];[Arg413Pro]  p.[IVS6-1G>A];[Ex6-96A>G]  p.[Leu242Phe];[Val388Met]  p.[Arg241Cys];[IVS7+2T>A]  p.[Arg157Lys];[Ex6-96A>G]  p.[Ser349Ala];[Arg241Cys]  p.[Ser70del];[Val388Met]  p.[Arg243Gln];[Ile324Asn]  p.[Arg243Gln];[Val388Met]  p.[Ex6-96A>G];[Arg241Cys]  p.[Arg243Gln];[Arg413Pro]  p.[IVS4-1G>A];[Arg241Cys]  p.[Gly257Val];[Val399Val]  p.[Ser70del];[Arg261Gln]  p.[Tyr166*];[Tyr166*]  p.[Ser70del];[Arg243Gln]  p.[Glu183Gly];[Arg241Cys]  p.[Arg270Ser];[Arg408Gln]  p.[Arg241Cys];[Glu286Lys]  p.[Ex6-96A>G];[IVS6-1G>A]  p.[Arg243Gln];[Arg243Gln]  p.[Phe121Leu];[Arg241Cys]  p.[Ex6-96A>G];[Arg243Gln]  p.[Glu280Lys];[Ala434Asp]  p.[Arg158Trp];[Val399Val]  p.[Arg241Cys];[Gly247Arg]  p.[IVS4-1G>A];[Tyr356*]  p.[IVS4-1G>A];[Arg413Pro]  p.[Gly257Val];[Arg408Gln]  p.[Arg243Gln];[Met276Lys]  p.[IVS4-1G>A];[Arg413Pro]  p.[Ex6-96A>G];[Arg243Gln]  p.[Arg241Cys];[Glu286Lys]  p.[Arg111*];[Val399Val]  p.[Arg241Cys];[Gly247Arg]  p.[Ala156Pro];[Arg408Gln] | **Age at the BH4**  **loading test**  ND  ND  ND  ND  ND  ND  ND  ND  ND  ND  ND  ND  ND  ND  2m  3m  ND  1m  4m  ND  ND  ND  2m  ND  ND  2m  2m  ND  ND  ND  2m  2m  2m  ND  ND  2m  2m  ND  ND  ND  ND  ND  ND  3m  ND  2m  4m  2m  3m  ND  ND  3m  ND  ND  3m  3m  ND  ND  ND  3m  ND  ND  2m  2m  ND  3m  4m  2m  3m  3m  2m  ND  3m  ND  ND  2m  2m  ND  ND  3m  ND  2m  ND  2m  ND  ND  3m  2m  ND  3m  ND  4m  3m  2m  2m  2m  3m  ND  ND  ND  ND  ND  ND  3m  2m  2m  ND  3m  2m  ND  3m  3m  4m  2m  ND  ND  3m  2m  ND  2m  3m  ND  2m  ND  2m  ND  2m  ND  4m  ND  3m  ND  ND  ND  3m  ND  ND  3m  ND  ND  ND  ND  ND  2m  ND  ND  ND  2m  ND  ND  ND  ND  ND  ND  3m  ND  2m  ND  ND  ND  ND  ND  ND  ND  ND  ND  ND  ND  ND  ND  ND  ND  ND  ND  ND  ND  ND  ND  ND  ND  ND  ND  ND  ND  ND  ND  ND  ND  ND  ND  ND  ND  ND  ND  ND  ND  ND  ND  ND  ND  ND  ND  ND  ND  ND  ND  ND  ND  ND  ND  ND  ND  ND  ND  ND  ND  ND  ND  ND  ND  ND  ND  ND  ND  ND  ND  ND  ND  ND  ND  ND  ND  ND  ND  ND  ND  ND  ND  ND  ND  ND  ND  ND  ND  ND  ND  ND  ND  ND  ND  ND  ND  ND  ND  ND  ND  ND  ND  ND  ND  ND  ND  ND  ND  ND  ND  ND  ND  ND  ND  ND  ND  ND  ND  ND  ND  ND  ND  ND  ND  ND  ND  ND  ND  ND  ND  ND  ND  ND  ND  ND  ND  ND  ND  ND  ND  ND  ND  ND  ND  ND  ND  ND  ND  ND  ND  ND  3m  ND  2m  2m  ND  ND  3m  ND  ND  3m  2m  4m  3m  ND  2m  ND  2m  ND  2m  3m  ND  3m  3m  ND  2m  ND  3m  2m  2m  2m  ND  2m  2m  2m  2m  5m  ND  2m  3m | **Data on BH4**  **response**  ND  ND  ND  ND  ND  ND  ND  ND  ND  ND  ND  ND  ND  ND  R  R  ND  R  R  ND  ND  ND  R  ND  ND  NR  NR  ND  ND  ND  R  R  NR  ND  ND  R  NR  ND  ND  ND  ND  ND  ND  R  ND  NR  R  NR  R  ND  ND  NR  ND  ND  R  R  ND  ND  ND  NR  ND  ND  NR  NR  ND  R  NR  R  NR  R  R  ND  R  ND  ND  NR  NR  ND  ND  NR  ND  NR  ND  NR  ND  ND  R  NR  ND  NR  ND  NR  NR  NR  NR  R  R  ND  ND  ND  ND  ND  ND  R  NR  NR  ND  R  NR  ND  NR  NR  NR  NR  ND  ND  NR  R  ND  NR  NR  ND  R  ND  NR  ND  NR  ND  R  ND  R  ND  ND  ND  NR  ND  ND  R  ND  ND  ND  ND  ND  NR  ND  ND  ND  NR  ND  ND  ND  ND  ND  ND  R  ND  NR  ND  ND  ND  ND  ND  ND  ND  ND  ND  ND  ND  ND  ND  ND  ND  ND  ND  ND  ND  ND  ND  ND  ND  ND  ND  ND  ND  ND  ND  ND  ND  ND  ND  ND  ND  ND  ND  ND  ND  ND  ND  ND  ND  ND  ND  ND  ND  ND  ND  ND  ND  ND  ND  ND  ND  ND  ND  ND  ND  ND  ND  ND  ND  ND  ND  ND  ND  ND  ND  ND  ND  ND  ND  ND  ND  ND  ND  ND  ND  ND  ND  ND  ND  ND  ND  ND  ND  ND  ND  ND  ND  ND  ND  ND  ND  ND  ND  ND  ND  ND  ND  ND  ND  ND  ND  ND  ND  ND  ND  ND  ND  ND  ND  ND  ND  ND  ND  ND  ND  ND  ND  ND  ND  ND  ND  ND  ND  ND  ND  ND  ND  ND  ND  ND  ND  ND  ND  ND  ND  ND  ND  ND  ND  ND  ND  ND  ND  ND  ND  ND  NR  ND  R  R  ND  ND  R  ND  ND  R  R  R  R  ND  R  ND  R  ND  R  R  ND  R  NR  ND  R  ND  NR  NR  R  R  ND  NR  R  NR  NR  R  ND  R  R |
| --- | --- | --- | --- | --- | --- | --- | --- |

ND not done; N/A Not available or not applicable R- responder; NR- non-responder

**Table S2 Characteristics of PAH deficient patients by groups (with BH4 loading test and without BH4 loading test)**

|  | | **with BH4 loading test** | | **without BH4 loading test** | **P** |
| --- | --- | --- | --- | --- | --- |
| **number of patients** | 94 | | 252 | | - |
| **male/female** | 40/54 | | 131/121 | | 0.06 |
| **age at diagnosis (days)**  **pre-Phe levels (uMol/L)*** | 18.1±9.4  1012.03±508.53 | | 23.6±10.2  1032.69±528.73 | | 0.7  0.15 |

*The maximum pretreatment Phe values.
